# Mechanical Valves for On‐Board Flow Control of Inflatable Robots

**DOI:** 10.1002/advs.202101941

**Published:** 2021-09-08

**Authors:** Lishuai Jin, Antonio Elia Forte, Katia Bertoldi

**Affiliations:** ^1^ John A. Paulson School of Engineering and Applied Sciences Harvard University Cambridge MA 02138 USA; ^2^ Department of Electronics Information and Bioengineering, Politecnico di Milano Milan 20133 Italy

**Keywords:** climbing, mechanical valves, robotic arm, rolling, soft robots

## Abstract

Inflatable robots are becoming increasingly popular, especially in applications where safe interactions are a priority. However, designing multifunctional robots that can operate with a single pressure input is challenging. A potential solution is to couple inflatables with passive valves that can harness the flow characteristics to create functionality. In this study, simple, easy to fabricate, lightweight, and inexpensive mechanical valves are presented that harness viscous flow and snapping arch principles. The mechanical valves can be fully integrated on‐board, enabling the control of the incoming airflow to realize multifunctional robots that operate with a single pressure input, with no need for electronic components, cables, or wires. By means of three robotic demos and guided by a numerical model, the capabilities of the valves are demonstrated and optimal input profiles are identified to achieve prescribed functionalities. The study enriches the array of available mechanical valves for inflatable robots and enables new strategies to realize multifunctional robots with on‐board flow control.

## Graphical abstract

This work presents several mechanical valves that aim at unloading the complexity of the robotic controls by embedding functionality into the design. Guided by a numerical model, the capabilities of the novel valves have been demonstrated by means of three robotic demos. This study enables new strategies to realize multifunctional robots with on‐board flow control.

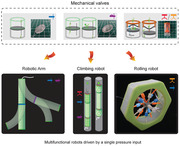

## 1 Introduction

From minimally invasive surgical tools^[^ ^1^, ^2^, ^3^, ^4^ ^]^ and assistive devices,^[^ ^5^, ^6^, ^7^, ^8^ ^]^ to compliant grippers^[^ ^9^, ^10^, ^11^, ^12^ ^]^ and video game add‐ons,^[^ ^13^, ^14^, ^15^ ^]^ inflatable soft robots have claimed an entire domain of applications for which safe interactions with the surrounding environment is the priority.^[^ ^16^, ^17^, ^18^, ^19^ ^]^ They are inherently compliant, easy to fabricate, and able to achieve complex motions harnessing the input pressure. However, the control of the fluid flow typically requires complex infrastructures comprising power sources, solenoid valves, electronic circuits, and pumps. As such, the development of strategies for an efficient actuation and control of inflatable soft robots is essential to the advancement of the field. Toward this end, various principles have been investigated to achieve flow control through pneumatic valves: flapping membranes inside the airways,^[^ ^20^, ^21^, ^22^ ^]^ collapsing soft channels,^[^ ^23^, ^24^, ^25^, ^26^, ^27^ ^]^ and kinks in soft tubes.^[^ ^28^, ^29^ ^]^ In addition, the snapping of bistable elastomeric membranes has recently been employed to design soft valves that use a second pressure signal to switch between states^[^ ^28^, ^30^, ^31^, ^32^ ^]^—the first of their kind to be built entirely from soft materials and to operate without the need of electronics. Such valves have demonstrated to provide a platform for the design of logic elements,^[^ ^30^ ^]^ ring oscillators that induce periodic motion using constant‐pressure,^[^ ^31^ ^]^ as well as electronics‐free pneumatic circuits for controlling soft‐legged robots.^[^ ^32^ ^]^ The design of mechanical valves that can be integrated into soft robots and operated without the needs of additional inputs is, however, still at an early stage. A more extensive library of elements would be ideal in order to expand the capabilities of soft robots that can operate without the need of electronic components.

Inspired by the recent progress in the design of mechanical valves, here we use different strategies to design and realize simple, easy to fabricate, lightweight and inexpensive mechanical valves, that are driven by the same pressure input used for the robot's actuation. Our designs harness both viscous flow and snapping arch principles, can be fully integrated on‐board and enable the control of the incoming airflow to realize multifunctional robots that operate with a single pressure input and no need for electronic components. These include a soft robotic arm capable of achieving multiple trajectories, a robot capable of climbing vertically in a tube carrying two times its own weight and even grasping an object and pulling it down, and a rolling robot that can successfully navigate in two directions.

## 2 Our Mechanical Valves

**Figure 1:**
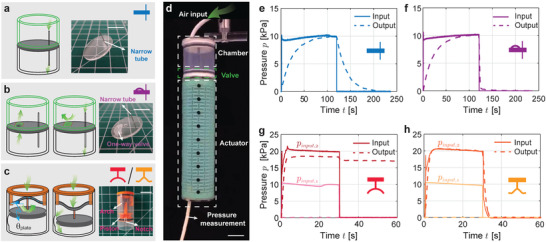
Design and characterization of the mechanical valves. a–c) Schematics and experimental snapshots of the valves. a) The viscous valve (indicated by the blue icon) consists of a acrylic plate and a narrow tube with length *l*_*tube*_ = 25 mm and inner diameter *d*_*tube*_ = 0.21 mm. b) The one‐way viscous valve (indicated by the purple icon) is a viscous valve with an additional one‐way gate which cause a pressure lag only in one direction. c) The hysteretic (indicated by the red icon) and bistable (indicated by the yellow icon) valves exhibit tunable mechanical responses controlled by a snapping arch connected to a movable piston. d) Experimental setup to test the valves. e–h) Pressure evolution at the inlet and outlet of each valve. e) Viscous valve: a delay in the output response in respect to the input is visible in inflation and deflation. f) One‐way viscous valve: a delay in the output response in respect to the input is visible only in inflation. g) Hysteretic valve: the on/off thresholds for this valve are ≈20 kPa and ≈2 kPa, respectively. h) The bistable valve only needs energy to switch its on/off state and would maintain that state once it actuated. Scale bars = 15 mm.

To simplify the flow control in soft robots, we design, realize, and test four fluidic mechanical valves. The first design, which we refer to as viscous valve, uses a narrow tube with length *l*_*tube*_ = 25 mm and internal diameter *d*_*tube*_ = 0.21 mm to provide a transient pressure difference between adjacent actuators (**Figure** **1**a) and causes their sequential activation. To demonstrate the capability of this valve we place it between a rigid chamber and a kirigami‐based soft actuator^[^ ^33^ ^]^ (Figure 1d). We then supply the chamber with air pressurized at *p*_*input*_ = 10 kPa for *t*_*input*_ = 120 s and monitor the pressure evolution inside the soft actuator, *p*_*output*_. As shown in Figure 1e, the valve introduces a transient pressure difference between the chamber and the actuator and that it takes ≈100 s for *p*_*output*_ to stabilize to *p*_*input*_ during both inflation and deflation (see Sections S1.1, S1.2, and S2.1, Supporting Information for additional details about design/fabrication of all our mechanical valves, fabrication of the kirigami actuator and testing, respectively).

The second design is similar to the first one, with the exception of an additional, larger hole (*d*_*hole*_ = 3 mm) placed next to the narrow tube. This aperture is covered on the top by a soft elastomeric membrane (10 mm long and 6 mm wide) fixed at the two opposite edges (Figure 1b). Such membrane acts like a gate: it can be bent out of plane by an upward airflow but it stays shut against a downward flow. Hence, as shown in Figure 1f, this design functions as a one‐way viscous valve and introduces a transient pressure difference only during inflation.

While our first two valves harness viscous flow, we also exploit the snapping and bistability of elastic arches to realize on/off valves. We first form an arch by buckling a metallic plate of length *l*_*plate*_ = 17.5 mm, width *w*_*plate*_ = 5 mm and thickness *t*_*plate*_ = 0.075 mm while constraining the rotation at its two ends. We then slide the ends of the buckled plate into two slits oriented at an angle *θ*_*plate*_ = 45° with respect to the horizontal direction situated at the opposite sides of a 3D‐printed plastic holder (Figure S1c, Supporting Information). Next, we connect the center of the buckled plate to a 3D‐printed piston, with head diameter of *d*_*disk*_ = 22 mm. Finally, we insert the system into a cylindrical plastic chamber with inner diameter equal to that of the piston head (i.e., *d*_*chamber*_ = *d*_*disk*_) to separate it into two volumes and place two O‐rings at the top of the holder and around the piston (Figure 1c), to ensure sealing during operation. Importantly, the chamber presents a small, dome‐shaped notch on its internal surface (with depth around 1 mm and diameter around 5 mm). Initially, when the arch is curved upward, the head of the piston is located above the notch. In this configuration the airflow is obstructed and *p*_*output*_ remains zero. As an example, if the input is a rectangular pulse with magnitude *p*_*input*, 1_ = 10 kPa, the valve remains closed and *p*_*output*_ = 0 (see light red line in Figure 1g). Differently, if *p*_*input*, 2_ ≈20 kPa the arch snaps and inverts its curvature, carrying the piston's head across the notch. In this second configuration the fluid in the two separate volumes of the chamber can exchange through the notch. However, the arch behaves hysteretically and snaps back when the pressure difference between the two sides of the piston is smaller than ≈2 kPa. As a consequence, the piston travels back across the notch, turning the valve off. This results in a steady pressure offset between the inlet and outlet. Even after the input pressure is removed, the pressure inside the kirigami actuator is kept at such steady level by the valve, so that the actuator remains inflated.

Further, by simply varying the mounting angle *θ*_*plate*_ to 0° one can transform the hysteretic valve into a bistable one, which switches state and remains open even when the input pressure is removed. To realize such bistable valve and have it operating at around 20 kPa, we reduce the thickness of the metallic plate to *t*_*plate*_ = 0.05 mm (while keeping *l*_*plate*_ = 17.5 mm and *w*_*plate*_ = 5 mm). This arch remains curved upwards for *p*_*input*_ < 20 kPa. Therefore, for low pressure inputs the measured outlet pressure is equal to zero (see results in Figure 1h for *p*_*input*, 1_ = 10 kPa). However, if the input pressure goes over *p*_*input*, 2_ = 20 kPa, the arch snaps to its inverted stable state and the valve opens (see dark orange line in Figure 1h). Importantly, our bistable valve remains in the opened state even after the input pressure is removed, resulting in a synchronous pressure variation between the input and output (i.e., once the input is removed, the output drops to zero at the same time). A large negative pressure impulse is needed to close this valve (see Figure S14, Supporting Information for additional details about the effect of geometric parameters on the snapping response of our hysteretic and bistable valves).

## 3 Robotic Arm with Different Trajectories

**Figure 2:**
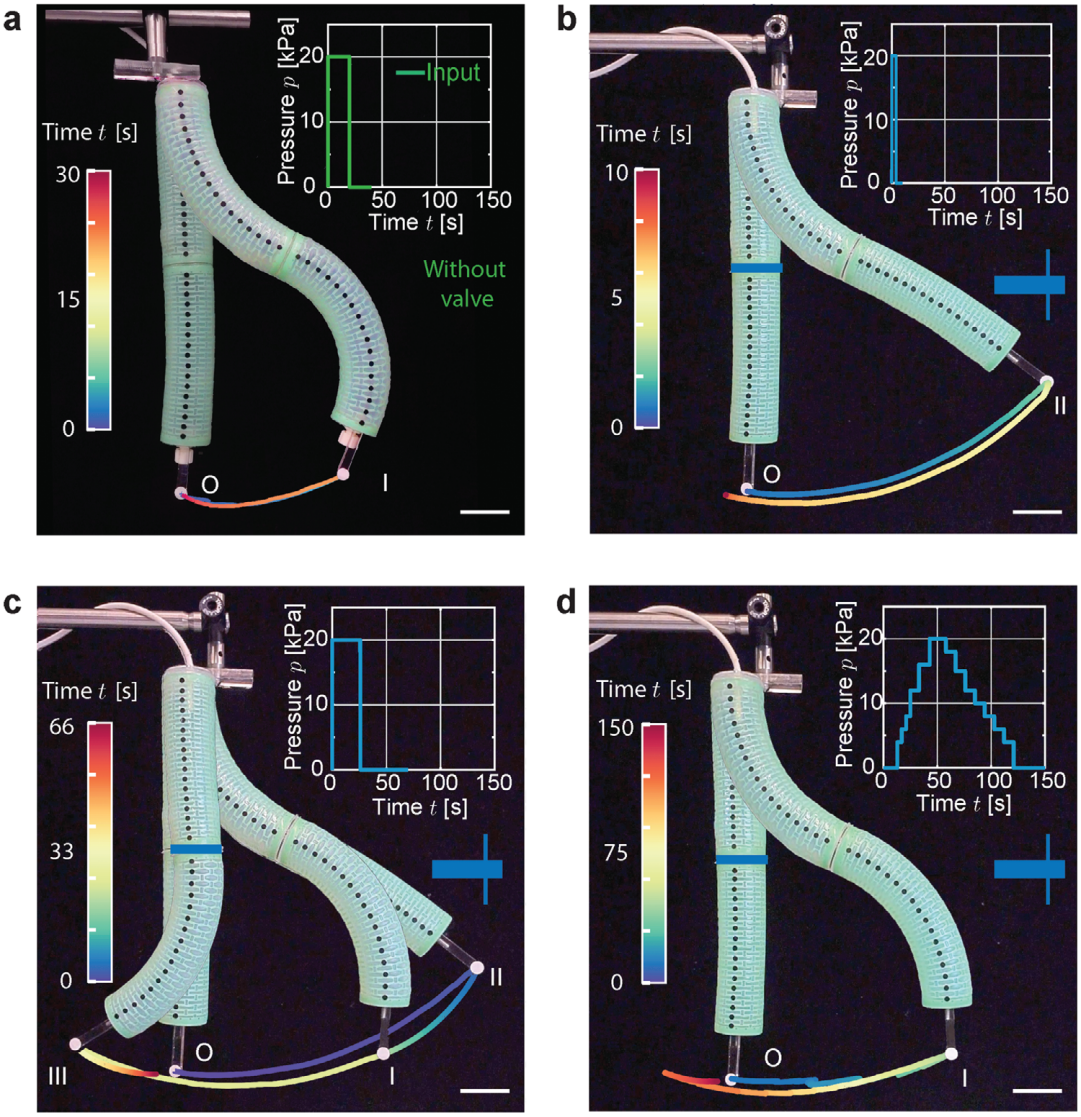
Robotic arm achieving different trajectories. a) A robotic arm comprising two bending actuators with no valve in between them bends to a S‐shape when a rectangular pressure pulse is applied (*p*_*input*_ = 20 kPa for *t*_*input*_ = 20 s). b–d) By introducing a viscous valve between the two actuators, the trajectory of the arm's tip can be regulated by varying the pressure‐time profile in input. b) The endpoint of this robotic arm moves further away compared to the previous version when pressurized (*p*_*input*_ = 20 kPa for *t*_*input*_ = 4 s), reaching position II (196 mm away from the initial position), and immediately back to the initial position when the pressure is removed. c) When a longer rectangular pressure pulse is fed to the robot (*p*_*input*_ = 20 kPa for *t*_*input*_ = 26 s) the end point of the robotic arm is able to reach three different positions. d) When undergoing to a gradually changed pressure, the robotic arm deforms to an S‐shape, resembling the same deformation of the arm without valve. Scale bars = 30 mm.

After introducing our four mechanical designs, we now show how to integrate them with several actuators and realize multifunctional robotic systems powered by a single pressure input. As a first example, we combine two bending kirigami actuators and a viscous valve to realize a robotic arm that can assume different trajectories by varying a single pressure input. As a reference, in the case of the two actuators simply connected without a valve in between, the robotic arm start straight and morphs into an S‐shape upon application of a rectangular pressure pulse (*p*_*input*_ = 20 kPa for *t*_*input*_ = 20 s, see inset in **Figure** **2**a). The actuator's tip reaches a point 102 mm away from the initial position when fully pressurized (I in Figure 2a) and goes back to its initial configuration when the pressure returns to 1 atm (Figure 2a). Clearly, for such robotic arm the deformation is only affected by the magnitude of *p*_*input*_. However, by introducing a viscous valve between the two actuators we can control the trajectory of the tip by modulating the pressure‐time profile in input (see Figure 2b–d). To demonstrate the concept, we power the new version of the robotic arm with a short rectangular pressure pulse (*p*_*input*_ = 20 kPa for *t*_*input*_ = 4 s, see the inset of Figure 2b). This results in the top actuator deforming instantaneously upon pressurization, whereas the presence of the viscous valve delays the activation of the bottom one. Therefore, the endpoint of this robotic arm moves further away compared to the previous version when pressurized (reaching position II, 196 mm away from the initial position) and immediately back to the initial position when the pressure is removed. If a longer rectangular pressure pulse is fed to the robot (*p*_*input*_ = 20 kPa for *t*_*input*_ = 26 s, see the inset of Figure 2c), the end point of the robotic arm is still able to reach position II during the first few seconds of the cycle. However, as time increases, the bottom actuator also bends, so that the robotic arm gradually mutates into the S‐shape configuration, and the endpoint moves to position I. Further, when the input pressure is removed (i.e., for *t*_*input*_ > 26s), the top and bottom actuators cannot deflate simultaneously due to the presence of the valve. Consequently, when the top actuator goes back into the initial, straight position, the bottom one is still bent: the endpoint reaches a new position III (65 mm apart from position O) on the left side of the initial configuration before returning to the rest position. Finally, a similar trajectory to that obtained in the absence of the viscous valve can be achieved, by gradually varying the pressure (*p*_*input*_ increases from 0 to 20 kPa in 50 s and then decreases to 0 kPa in 100 s—Figure 2d). Note that in this case a left swing is observed at the end of the cycle. This is due to the step‐like nature of the pressure input, and can be eliminated by using a smoother pressure input (see Sections S1.3 and S2.3, Supporting Information for additional details about fabrication and testing of the robotic arm).

Whereas in Figure 2 we consider a robotic arm with a viscous valve, the range of achievable trajectories can be further enlarged by incorporating a one‐way viscous valve (Figure S17, Supporting Information) and connecting more than two actuators (Figures S18 and S19, Supporting Information). Finally, the behavior of the robotic arm under different pressure inputs can be nicely captured by a simple numerical model based on the Navier–Stokes equations,^[^ ^34^ ^]^ expanded to take into account air compressibility (see Section S3.1, Supporting Information for additional details about the model).

## 4 Tube Climbing Robot

Thus far, we have demonstrated the capabilities of individual valves (viscous and one‐way viscous valve) for the realization of a robotic arm with tip trajectories that can be prescribed by modulating the input pressure. As second demonstration we combine different valves together and realize a robot capable of climbing vertically in a pipe. Moreover, the robot can carry twice its own weight and even grasp an object. Differently from previous tube‐climbing robots,^[^ ^35^, ^36^, ^37^, ^38^ ^]^ our system operates with a single pressure input.

**Figure 3:**
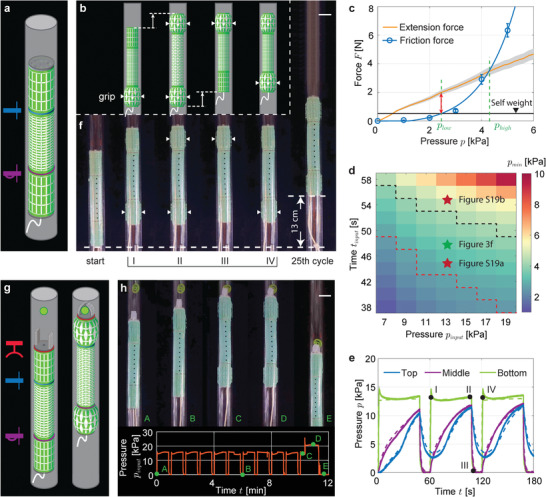
Multifunctional climbing robot. a) Schematic of the climbing robot comprising three kirigami actuators (two expanding and one extending actuator), a one‐way viscous valve and a viscous valve. b) Climbing of the robot is enabled by the sequential inflation and deflation of the actuators, which can be modulated by varying the pressure‐time profile of a single pressure input. c) The evolution of the friction between the pipe and the expanding actuator as well as the extension force generated by the middle actuator, as a function of the internal pressure. According to the plots, the minimum pressure inside the expanding top actuator should be larger than *p*_*low*_ = 2.5 kPa to prevent slipping and smaller than *p*_*high*_ = 4.2 kPa to enable axial extension toward the top. Error bars indicate the standard deviation for the measurements. d) Phase diagram of the minimum pressure inside the top actuator (after the first cycle). The area of the diagram that is bounded by the two dashed lines identifies the input parameters for which the robot will achieve climbing. e) Comparison between the experimental and numerical results of the pressure evolution inside the actuators. The good agreement between them enables one to predict the behavior of the robot using the numerical model. f) Experimental demonstration of the climbing robot which climbs 13 cm in 25 cycles with *p*_*input*_ = 13 kPa, *t*_*input*_ = 48 s and *T* = 60 s. g) Schematic of the climbing robot capable of grasping an object and pulling it down, which is realized by adding an additional hysteretic valve and gripper to the top actuator. h) Experimental snapshots of the climbing robot with the gripper. Scale bars =30 mm.

To realize such robot we connect two expanding kirigami actuators to an extending one via a one‐way viscous valve and a viscous valve (**Figure** **3**a). Upon pressurization, the bottom actuator expands instantaneously and grips onto the pipe (Figure 3b). At the same time the top and middle actuators deform gradually due to the presence of the viscous valves. This results in the head of the robot moving upwards before the top actuator is able to grip onto the pipe. As the pressure input is removed, the top actuator remains fully inflated, due to the lag introduced by the viscous valve, and acts as anchoring point. Differently, due to the presence of the one‐way viscous valve which offers negligible resistance for the air to instantaneously escape, the middle and bottom actuators deflate synchronously. With the top actuator still anchored, the deflation translates in an upward movement for the lower part of the robot, which closes the loop.

Note that such deformation sequence is promising toward enabling a climbing motion, since it is asymmetric and makes the robot extend upwards. However, it only translates into vertical climbing if the frictional force between the inflated top actuator and the tube is i) larger than the weight of the robot (or it will fall) and ii) smaller than the axial extension force generated by the middle actuator (or it will not extend upwards). Since the friction and extension force are dependent on the pressure in each actuator, a tailored input pressure signal is crucial for the ability of the robot to climb. In order to identify input signals that enable climbing, we first measure i) the evolution of the friction between the pipe and the expanding actuator and ii) the extension force generated by the middle actuator as a function of the internal pressure. As shown in Figure 3c, we find that the minimum pressure inside the expanding top actuator should be larger than *p*_*low*_ = 2.5 kPa to prevent slipping and smaller than *p*_*high*_ = 4.2 kPa to enable axial extension toward the top.

To identify a suitable input signal that satisfy these requirements, we run 77 numerical analysis where we systematically vary *p*_*input*_ and *t*_*input*_ (with *p*_*input*_ ∈ [7, 19] kPa and *t*_*input*_ ∈ [38, 58] s) while keeping the actuation period equal to *T* = 60 s. In Figure 3d, we report the minimum pressure recorded for the top actuator after the first cycle, *p*_*min*_, as a function of *p*_*input*_ and *t*_*input*_. Within the explored design space we find a ”climbing” region (bounded by the two dashed lines) for which *p*_*min*_ is greater than *p*_*low*_ and smaller than *p*_*high*_. To validate these numerical predictions, we conduct experiments in which we provide an input pressure of *p*_*input*_ = 13 kPa and select *t*_*input*_ guided by the results of Figure 3d. Note that in order to maximize the extension toward the top of the middle actuator and, in turn, increase the efficiency of the robot, for a given value of *p*_*input*_, *t*_*input*_ should be selected to be as close as possible to the lower boundary of the ”climbing” region. In fact, the closer we operate to the lower boundary, the greater is the difference between the vertical extension force exerted by the middle actuator and the friction between top actuator and the tube (Figure 3c, red segment). As such, we first choose *t*_*input*_ = 45 s (Figure S20a, Supporting Information). However, because of small discrepancies between our experiments and model (which slightly over predicts the minimum pressure in the top actuator—Figure 3e), we find that the frictional force is not enough to prevent falling. Therefore, we increase *t*_*input*_ to 48 s to move slightly away from the lower boundary of the domain and find that for this input the robot climbs upwards and moves of 13 cm in 25 cycles (Figure 3f). In Figure 3e, we report the resultant input pressure profile fed into the bottom actuator of the robot (green curve), along with the pressure profiles measured in all the chambers (continuous lines) and those predicted by the model (dashed lines) and find very good agreement between the two sets of data. Further, these results are in agreement with the predicted climbing sequence shown in Figure 3b. In fact, when *p*_*input*_ is removed, the pressure decreases in all three actuators. However, while this drop is almost instantaneous in the bottom and middle actuator, it is less sharp in the top one due to the delay introduced by the viscous valve. Importantly, this results in a non‐zero minimum pressure for the top actuator, which enables upward retraction of the other two chambers while assuring anchoring. Additionally, it is worth noticing that if *t*_*input*_ is further increased to 55 s, the robot does not move upwards as the frictional force between the top actuator and the pipe becomes too large (Figure S20b, Supporting Information). Further, we note that, using the same approach, the input signal can be also optimized to make the robot carrying two time its weight (Figure S20d and Movie S3, Supporting Information). Finally, we want to emphasize that the slow speed of the robot is due to the low flow rate of our input pump (ITV1030 by SMC Cooperation, with flow rate around 7.5 mL s^−1^ at pressure of 20 kPa) rather than our mechanical valves and that the speed could be largely increased by using a better input pump to increase the flow rate (Figure S22, Supporting Information).

Next, to enhance the functionality of the robot and making it capable of grasping an object and pulling it down, we add a gripper to its head (comprising two PneuNets^[^ ^9^ ^]^) and connect it to the top actuator via an hysteretic valve (Figure 3g). Since the valve is embedded into the top actuator, it affects the actuator's pressure‐volume relationship and, in turn, alters the input parameters required for climbing. Therefore we rerun the numerical analysis accounting for the internal volume reduction of the top actuator and find that larger values of *p*_*input*_ are required to achieve locomotion (Figure S21, Supporting Information). Guided by the numerical analyses we then choose *p*_*input*_ = 15 kPa and *t*_*input*_ = 48 s. As shown in Figure 3h, we find that, when *p*_*input*_ = 15 kPa is cyclically applied, the robot navigates upwards toward the object. Then, as the robot approaches the object, a pressure higher than the threshold pressure of the hysteretic valve (*p*_*input*_= 22 kPa) is supplied (the input pressure profile for the experiments is reported in the bottom panel of Figure 3h). The valve opens and actuate the gripper, which grasps the object in the pipe. Afterwards, when the pressure in the gripper approaches the pressure inside the top actuator, the hysteretic valve snap back, switching off. At this point, the gripper remains inflated, keeping hold of the object. Finally, the three kirigami actuators can be deflated to remove the anchoring points and let the robot slide down with the captured object.

## 5 Rolling Robot

**Figure 4:**
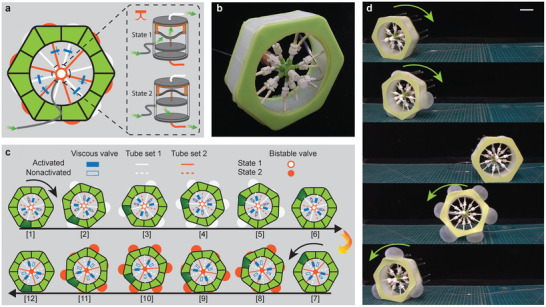
Rolling robot. a) A hexagonal rolling robot integrated with a modified version of the bistable valve and six viscous valves is able to roll bi‐directionally with a single pressure input. The bistable valve has two states, which allow it to dispense the input flow into two separate circuits, each connecting six chambers (highlighted in white and orange). Each circuit has three chambers connected directly to the bistable valve, while other three chambers are connected to the bistable valve through viscous valves. b) Experimental snapshots of the rolling robot. c) Operating principle of the rolling robot. The chambers on the rolling robot inflate/deflate sequentially due to the presence of viscous valves pushing the robot away from one face to another. The bistable valve, which can be switched by applying a pressure burst, dispenses the input flow into different circuits enabling the bi‐directional rolling. d) Experimental snapshots of the robot rolling in both directions. Scale bars = 50 mm.

To further illustrate the capability of our valves, we design and test a hexagonal rolling robot integrated with one bistable valve and six viscous valves. This robot is different from other rolling robots^[^ ^31^, ^39^, ^40^, ^41^, ^42^ ^]^ due to its ability to roll bi‐directionally with a single pressure input. The robot comprises twelve inflatable chambers evenly distributed along the perimeter of an hexagon (two on each side, see **Figure** **4**a,b). Such chambers act as legs and by selectively inflating and deflating them we can push the robot away from a stable equilibrium and make it roll forward or backward.

Such rolling motion can be realized with a single input pressure by introducing viscous valves. More specifically, we form a circuit connecting six inflatable chambers and the input source (white connections in Figure 4a). Three of these chambers are connected directly to the pressure supplier. Alternating these, there are three additional chambers, each of which is connected to a viscous valves before being connected to input (see schematics in Figure 4a). When a pressure input is supplied, the chambers directly attached to the input valve inflate first (snapshot 2 in Figure 4c). This unbalances the robot to the point that it rolls over the hexagon's corner and lay flat on an edge where the chamber is still deflated (snapshot 3 in Figure 4c). At this point the chambers attached to the viscous valves, which suffer an inflation delay, start to inflate too (snapshot 4 in Figure 4c). When the input pressure is removed, the chambers with no viscous valve deflate instantaneously, while the ones with viscous valves keep the inflated state temporarily and push the robot to rotate further (snapshot 5 in Figure 4c) until all the chamber deflate to the initial state (snapshot 6 in Figure 4c). The robot can keep rotating forward by repeating the aforementioned procedure. Note that, in an effort to speed up the locomotion, in this demonstration the diameter of the viscous valves's narrow tube was increased to *d*_*tube*_=0.26 mm.

Further, the direction of rolling can be changed by introducing an identical circuit that connects the remaining six chambers and a slightly modified design of the bistable valve in which the notch is replaced with a hole with diameter of 1.6 mm. When the input source is connected to this hole (see schematics in Figure 4a), the new version of the bistable valve is able to switch the flow from one circuit to the other (highlighted in white and orange in Figure 4a). This is possible by applying a negative pressure burst to the input to snap the arch and, in turn, change the position of the piston. The piston travels over the hole and switches the valve's output to the second circuit (orange tubes), where the inflatable chambers are positioned in the opposite order. The robot can then start rotating in the opposite direction by repeating the same inflation cycle (snapshots 7–12 in Figure 4c).

To demonstrate the concept experimentally we fabricate a prototype out of elastomeric materials by means of a molding approach. From the experimental snapshots in Figure 4d, we can see that the robot rolls from one face of the hexagonal frame to the next as the chamber on each face sequentially inflate and deflate. Further, by applying a negative pressure pulse (*p*_*input*_ = −25 kPa, see Figure S11 and Movie S4, Supporting Information) we can switch the state of the bistable valve and change the direction of rolling.

## 6 Conclusion

In summary, we have designed and built simple, easy to fabricate, lightweight, and inexpensive mechanical valves that can be easily integrated with soft actuators to control the airflow and realize robotic systems that operate with a single pressure input. Our mechanical valves provide new opportunities to realize sequential operation robots, and enrich the array of existing valves. Specifically, while the large majority of the existing designs require an external input to operate—including soft bistable valves,^[^ ^28^ ^]^ thermal responsive microvalves,^[^ ^20^ ^]^ and microfluidic devices^[^ ^23^, ^24^ ^]^—our valves are fully passive and easy to integrated on‐board, facilitating the design of robots that can selectively activate different parts, depending on the application. Further, while most previously proposed designed are on/off valves,^[^ ^25^, ^28^, ^29^ ^]^ our viscous valves can generate flow resistance in symmetric or asymmetric ways, enabling the design of robots with sequential operation. While in this study we have demonstrated the potential of our valves by using kirigami fluidic actuators, our devices can interface with any type of pneumatic soft actuator to control the incoming airflow and realize robots that operate with a single pressure input. Further, besides the three robotic demos presented here, our devices hold potential to be employed in various robotic systems in the future. For example, by integrating a viscous and hysteretic valve together, we could design a new valve that offers a pressure lag when supplying low pressure (lower than the threshold of the arch snapping), while exhibits instant on/off switch when supplying higher pressure. This might enable the design of biomimicking robots capable of switching between different motion modes (e.g., walking and jumping), despite a single input pressure. Finally, one could expand the current study by investigating the behavior of complex systems comprising valves in series and parallel configurations and enrich the array of available valves with new designs harnessing instabilities in compliant shell elements.

## Conflict of Interest

The authors declare no conflict of interest.

## Supporting information

Supporting Information

Supplemental Movie 1

Supplemental Movie 2

Supplemental Movie 3

Supplemental Movie 4

## Data Availability

The data that support the findings of this study are available from the corresponding author upon reasonable request.
