# Supplementary material for: Mechanical Valves for On‐Board Flow Control of Inflatable Robots
Source: Adv Sci (Weinh). 2021 Sep 8;8(21):2101941. doi: 10.1002/advs.202101941 (PMC8564437; doi:10.1002/advs.202101941)
Supplement: Supplementary file 1 — Supporting Information [file ADVS-8-2101941-s002.pdf]

© 2021 Wiley-VCH GmbH

ADVANCED SCIENCE Open Access

Supporting Information

for *Adv. Sci.*, DOI: 10.1002/advs.202101941

Mechanical Valves for On-Board Flow Control of Inflatable Robots

*Lishuai Jin, Antonio Elia Forte, and Katia Bertoldi**

Supporting Information for

Mechanical valves for on-board flow control of inflatable robots

Lishuai Jin, Antonio Elia Forte, and Katia Bertoldi

E-mail: bertoldi@seas.harvard.edu

This PDF file includes:

Figs. S1 to S22
Tables S1 to S2
Captions for Movies S1 to S4
References for SI reference citations

Other supplementary materials for this manuscript include the following:

Movies S1 to S4

1. Fabrication

In this Section we provide details on the fabrication of our mechanical valves as well as the three robotic systems (i.e. the robotic arm, the climbing robot and the rolling robot)

1.1. Mechanical valves. To simplify the flow control in soft robots, we introduce four fluidic mechanical valves: (*i*) viscous valve, (*ii*) one-way viscous valve, (*iii*) hysteretic valve and (*iv*) bistable valve.

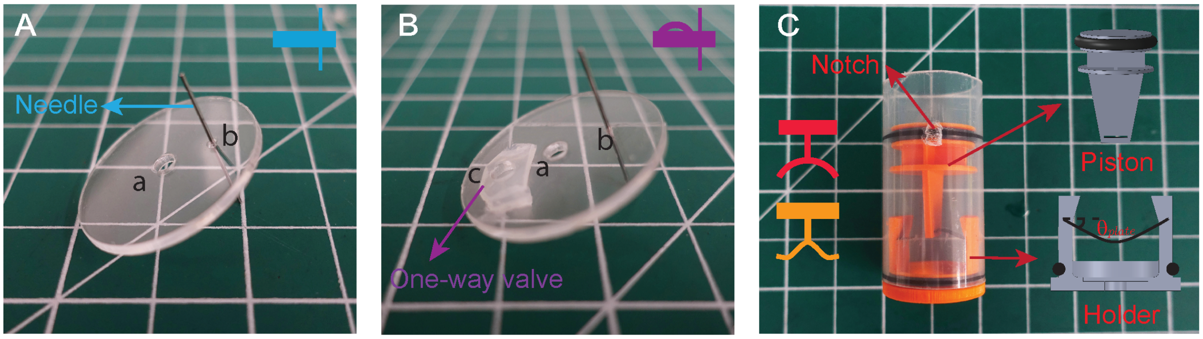

Fig. S1. Design of the mechanical valves. Experimental snapshots of (*A*) viscous valve, (*B*) one-way viscous valve and (*C*) hysteretic or bistable valve.

- *Viscous valve:* The viscous valve tested in this study comprises a circular acrylic plate with embedded a hollow tube with small diameter (Fig. S1*A*). Specifically, our viscous valves are fabricated using the following steps
  - Laser cut a circular acrylic plate with diameter of 30 mm and thickness of 1/16 inch.
  - Cut two holes in the acrylic plate: (**a**) one at the center with diameter of 3 mm that is used to connect adjacent actuators via a bolt; and (**b**) one with smaller diameter of ~ 0.8 mm that is used to embed the narrow tube.
  - Insert a narrow tube through the hole **b** of the acrylic plate and seal the gap between the tube and the hole using glue (Sil-Poxy Silicone Adhesive, Smooth-On). The narrow tubes used in this study are cut from glue needles (FEITA) and have length of 25 mm and inner diameter varying from 0.16 mm to 0.51 mm.
- *One-way viscous valve:* To obtain a valve with unidirectional pressure drop, we add a one-way valve to the viscous valve described above. Our one-way valves are fabricated using the following steps (Fig. S1*B*):
  - Cut another hole (with diameter of 3 mm) on the acrylic plate of the viscous valve (denoted with c in Fig. S1*B*). To avoid conflict when assembling the valve with actuators, this hole is in a diametrically opposite position to the one used for the needle.
  - Cast a rectangular shape membrane using Ecoflex 00-50 with length of ~ 10 mm, width of ~ 6 mm and thickness of ~ 1 mm.
  - Glue the two short edges of the membrane on the acrylic plate to cover hole **c**. If the membrane is attached to the top of the plate, it allows only flow from the bottom to the top. In fact the flow bends the membrane and allows the air through hole **c**.

Please note that when the valves are integrated with the actuators, the hole in the center of the acrylic plate should be sealed by an appropriate sealing ring to avoid air leaking (see Fig. S3 and S4).

- *Hysteretic valve:* Our hysteretic valve exploits the snapping of elastic arches (Fig.S1 C). To fabricate the valve, we follow the steps below:
  - Laser cut rectangular plates with width $w_{plate} = 5$ mm and length $l_{plate} = 17.5$ mm out of 1095 spring steel shims (McMaster-Carr product ID: 9503K31) with thickness $t_{plate} = 0.075$ mm and Young's modulus $E = 170$ GPa.
  - Use an Ultimaker S3 3D printer to fabricate an holder out of PLA material with two slits oriented at an angle $\theta_{plate} = 45°$ with respect to the horizontal direction
  - Apply an axial force to buckle the rectangular plate into an arch and slide its ends into the two slits of the 3D-printed holder and glue the arch to the slits using ethyl 2-cyanoacrylate glue (Krazy Glue, NC).
  - 3D print a piston and glue the piston at the center of the arch using Sil-Poxy (Smooth-On).
  - Cut a segment with length of ~ 40 mm out of a 10 ml plastic syringe with inner diameter of 22 mm. This will form the chamber for the valve.
  - Cut a dome-shaped notch (with depth around 1 mm and diameter around 5mm) on the internal surface of the cylindrical segment using a rotary tool workstation (220-01 Dremel). When the piston moves across the notch, the on/off state of the valve is changed.
  - Mount O-rings (Oil-Resistant Soft Buna-N, McMaster-Carr product ID: 2418T138) on the piston and the holder to prevent leaking between the piston (or holder) and the chamber.
  - Insert the construction of holder, arch and piston into the chamber.
- *Bistable valve:* The bistable valve is fabricated following the same steps used for the hysteretic valve. The only differences between the two designs are the thickness of the plate (for the bistable valve we use a metallic plate with thickness $t_{plate} = 0.05$ mm) and the mounting angle θ_{plate} (for the bistable valve we use $\theta_{plate} = 0°$).

1.2. Kirigami actuators. To realize the robotic arm and climbing robot shown in Fig. 2 and 3 of the main text, we use cylindrical kirigami actuators fabricated following the procedure described in our previous work (1) - the only difference is that here we add another thin layer (thickness $\sim$ 1 mm) of Ecoflex 00-50 inside the actuator to enhance its robustness and durability. Specifically, we use three types of actuators which support bending (Actuator I in Fig. S2), extension (Actuator II in Fig. S2) and expansion (Actuator III in Fig. S2). The geometric parameters defining each actuator are shown in Fig. S2 and listed in Table S1. Note that Actuators II and III comprise identical units for the entire structure, whereas to enable bending in Actuator I we introduce one column of unit cells with increased ligament size δ_2 (shown as purple units in Fig. S2).

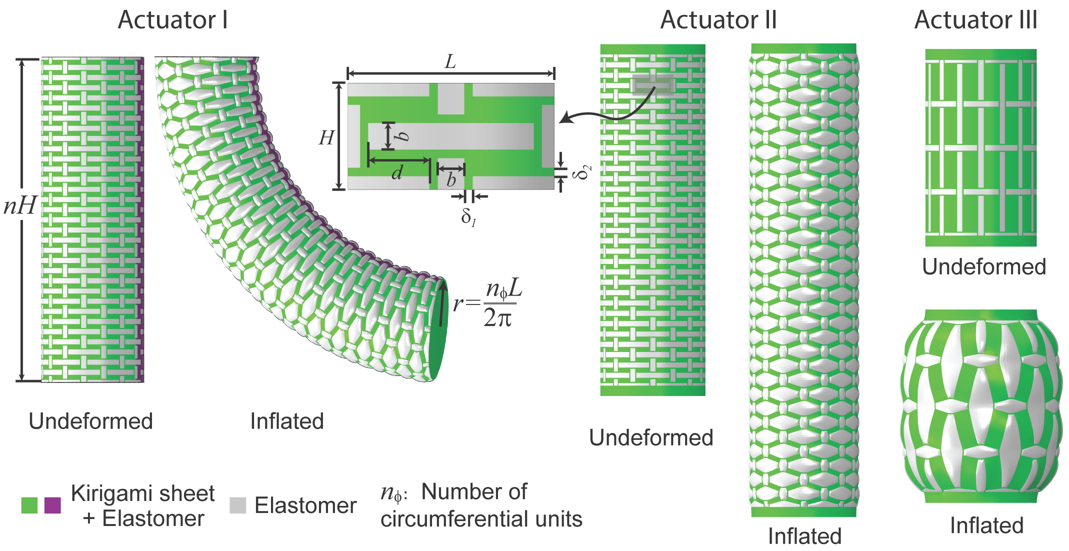

Fig. S2. Kirigami actuators. Three types of kirigami actuators are used in realizing bending (Actuator I), extension (Actuator II) and expansion (Actuator III) deformation. The geometric parameters of the actuators are listed in Table S1.

Geometric parameters defining the actuators							
Geometric parameters	L [mm]	H [mm]	b [mm]	δ_1 [mm]	δ_2 [mm]	n	n_ϕ
Actuator I (purple units)	12	6	3	0.36	2.16	16	1
Actuator I (green units)	12	6	3	0.36	0.36	16	7
Actuator II	12	6	3	0.36	0.36	16	8
Actuator III	12	24	3	0.36	0.36	2	8

Table S1. Geometric parameters defining our kirigami actuators. All parameters are defined in Fig. S2.

1.3. Robotic arm. The robotic arm is constructed by connecting two bending kirigami actuators (Actuator I in Table S1 and Fig. S2) through a viscous valve or a one-way viscous valve. Specifically, we fabricate the robotic arm using the following steps:

- Fabricate two identical kirigami actuators (Actuator I in Table S1 and Fig. S2) following the procedure described in our previous work (1). The only difference is that here we add another thin layer (thickness $t \sim 1$ mm) of Ecoflex 00-50 inside the actuator to enhance its robustness and durability. More specifically, we pour additional Ecoflex inside the kirigami actuator and rotate it slowly to achieve a uniform coating.

- Glue perforated acrylic plates at the ends of the actuator to facilitate connection with other actuators (see Fig. S3). Further, we 3D print a mold to cast elastomeric layers with thickness ~ 1 mm and the same shape as the acrylic plates. Such elastomeric layers are placed between the acrylic plates and the valves to avoid leakages (see Fig. S3).

- Arrange the two identical bending actuators so that the columns of unit cells with increased ligament size δ_2 are diametrically opposed (Fig. S3).

- Introduce a viscous valve (or one-way viscous valve) between the two actuators to regulate the flow between them (as shown in Fig. S3). Secure the connection between the actuators and the valve by threading a screw through the acrylic plates and the hole places in the center of the valve.

- Connect a tube to the top actuator to provide the pressure input.

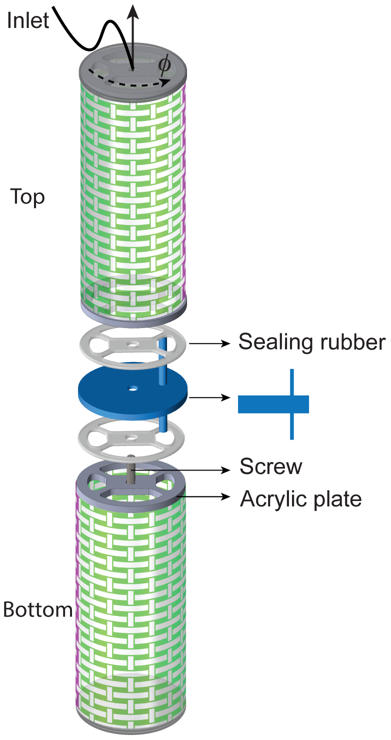

Fig. S3. Schematic of the robotic arm. The robotic arm is constructed by connecting two bending kirigami actuators (Actuator I in Table S1 and Fig. S2) separated by a viscous valve or one-way viscous valve.

1.4. Tube climbing robot. As shown in Fig. S4*A* our climbing robot comprises two expanding kirigami actuators (Actuator III in Table S1) and an extending one (Actuator II in Table S1) connected via a one-way viscous valve and a viscous valve. Further, to enable grasping, a hysteretic valve and a gripper consisting of two PneuNets (2) is added at the top (Fig. S4*B*). To fabricate the robot, we follow the steps below:

- Fabricate two identical expanding kirigami actuators (Actuator III in Table S1 and Fig. S2) and one extending actuator (Actuator II in Table S1 and Fig. S2) following the procedure described in our previous work (1). The only difference is that here we add another thin layer (thickness $t \sim 1$ mm) of Ecoflex 00-50 inside the actuator to enhance its robustness and durability. More specifically, we pour additional Ecoflex inside the kirigami actuator and rotate it slowly to achieve a uniform coating.
- Glue perforated acrylic plates at the ends of the actuators to facilitate connection with other actuators (see Fig. S4*A*). Further, we 3D print a mold to cast elastomeric layers with thickness $\sim$ 1 mm and the same shape as the acrylic plates. Such elastomeric layers are placed between the acrylic plates and the valves to avoid leakages (see Fig. S4*A*).
- Introduce a viscous valve between the top extending actuator and the elongating one and a one-way viscous valve between the bottom extending actuator and the elongating one (as shown in Fig. S4). Secure the connection between the actuators and the valve by threading a screw through the acrylic plates and a hole places in the center of the valve.
- Connect a tube to the bottom actuator to provide the pressure input.

Additionally, to realize a robot capable of grasping an object, the following steps are also needed:

- 3D print the two-part mold shown in Fig. S5*A*. This mold enables casting of PneuNets with length $l_{gripper} = 30$ mm, width $w_{gripper} = 10$ mm and thickness $t_{gripper} = 5$ mm.
- Prepare Ecoflex 00-30 and Ecoflex 00-50 to cast the PneuNets.
- Pour Ecoflex 00-30 inside the main body mold; pour Ecoflex 00-50 inside the base mold up to 2/3 of its height ($\sim$ 2 mm, Fig. S5*B*).
- Cure the Ecoflex for about an hour at room temperature.
- Remove the cured Ecoflex 00-30 from the main mold
- Apply a layer (with thickness of $\sim$ 0.5 mm) of uncured Ecoflex 00-50 on top of the cured Ecoflex 00-50 (while keeping the part in the base mold)
- Slowly place the Ecoflex 00-30 structure obtained from the main mold on top of the base mold. The uncured Ecoflex 00-50 enables bonding between the two pieces.
- Remove the soft actuator from the mold.
- Insert a short tube in one end of the PneuNets.
- Laser cut a circular acrylic plate with diameter of 30 mm and thickness of 1/16 inch.

- Cut three holes (with diameter of 3 mm) in the acrylic plate: (**a**) the one at the center is used for the connection with the top actuator via a screw; and (**b**) the other two holes, located at the margin of the plate, are used to pass through the short tubes that connect to the Pneunets.
- Apply glue (Sil-Poxy Silicone Adhesive, Smooth-On) to secure the tube and Pneunets to the acrylic plate.
- Attach a hysteretic valve to the top expanding actuator and connect the gripper to its chamber. Apply glue (Sil-Poxy Silicone Adhesive, Smooth-On) to fix and seal the valve and the gripper.
- The robot is ready to be tested.

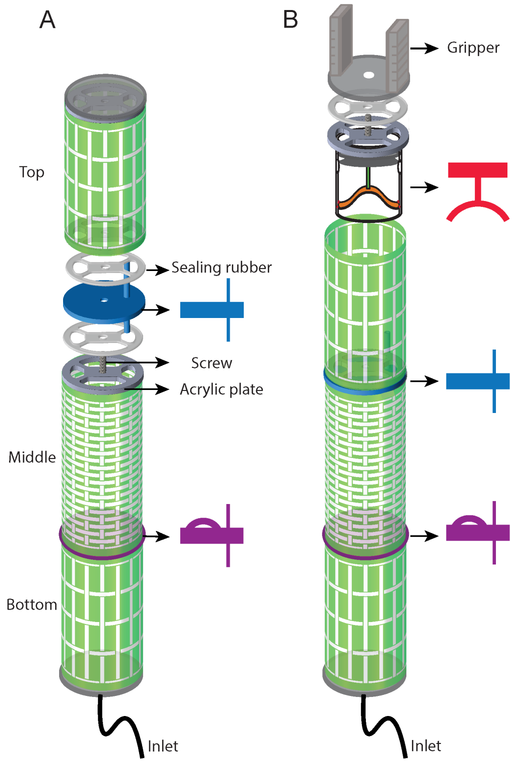

Fig. S4. Schematic of the climbing robot. (*A*) The climbing robot comprises two expanding kirigami actuators (Actuator III in Table S1) and an extending one (Actuator II in Table S1) connected via a one-way viscous valve and a viscous valve. (*B*) A hysteretic valve and a gripper consisting of two PneuNets (2) is added to the top actuator to enable grasping.

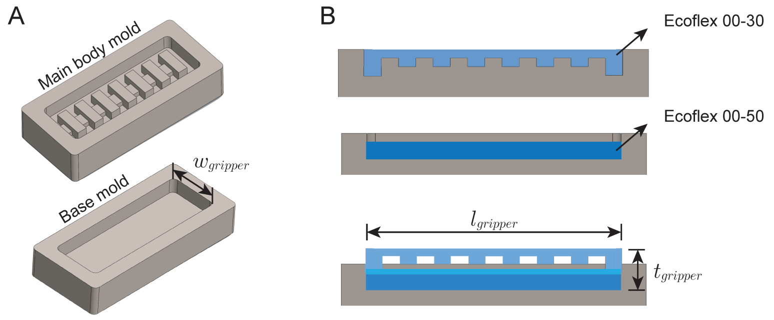

Fig. S5. Fabrication of the gripper. (*A*) 3D printed molds for the gripper. (*B*) Fabrication procedure.

1.5. Rolling robot. The rolling robot demonstrated in this study is made of nearly incompressible silicon rubbers. Specifically, we use Elite Double 32 (with green color and initial shear modulus $\mu = 0.35$ MPa, Zhermack) for the hexagonal frame of the robot and Ecoflex 00-50 (with white translucent color and initial shear modulus $\mu = 0.0405$ MPa, Smooth-On, PA) for the deformable chambers. 12 chambers are evenly distributed along the perimeter of the robot (two on each side), which are connected to the pressure source via six viscous valves and a modified bistable valve. The robot is fabricated using a molding approach according to the following 8 steps (Fig. S6):

- **Step A-1:** 3D print the mold shown in Fig. S6*A* using PLA material with a Ultimaker S3 printer. We insert screws inside the mold to facilitate the connection of the tubes in *Step G*.
- **Step A-2:** pour Ecoflex 00-50 into the mold to cast the skeleton of the chambers and let it cure for 30 minutes at room temperature. Note that the height of the Ecoflex should not be higher than that of the mold's islands shown in Fig. S6*A*.
- **Step B:** pour Elite Double 32 into the mold (on top of the cured Ecoflex 00-50) to cast one side of the frame.
- **Step C:** wait for Elite Double 32 to cure for 30 minutes.
- **Step D:** remove the cured Ecoflex 00-50 and Elite Double 32 from the mold. Note that this two materials bond naturally.
- **Step E:** use another mold to cast the other hexagonal frame of the robot, pour Elite Double 32 into the mold until the thickness is about 4/5 of the mold's thickness.
- **Step F:** when the Elite Double 32 frame is half cured (after ~15 minutes), pour more Elite Double 32 onto the half cured skeleton (to reach the full thickness) and then place the structure from *Step d* on top of it to seal the frame and the chambers.
- **Step G:** connect two sets of tubes to the robot to form two circuits. Each set has six tubes attached to the six chambers (one at each side of the robot). Three tubes in each set are equipped with a viscous valve. Note that to facilitate the (dis)assembling of the valves from the robot, we split each tube into two segments and attach them using a luer lock (with internal diameter 1/8 inch). We then insert the viscous valves into the luer lock. Note that in this case, we glue and seal the narrow needle (with length $l_{tube} = 25$ mm and diameter $d_{tube} = 0.26$ mm) of the viscous valve directly into the luer lock using Sil-poxy).
- **Step H:** Connect the two sets of tubes via a bistable valve (see the connection in Fig. S7).
- Modify the design of the bistable valve to enable bi-directional rolling. More specifically, we replace the notch with a hole with diameter of 1.6 mm. When the input source is connected to this hole (Fig. S7*A*), the new version of the bistable valve is able to switch the flow from one circuit to the other. This is possible by applying a negative pressure burst to the input to change the position of the piston and snap the arch at the same time, which forces the piston to stay in the new position. The connection between the bistable valve and viscous valves is shown in Fig. S7*B*.
- Add additional acrylic rods to one face of the robot to stabilize it and prevent it to fall sideways during the test (see Fig. 4*D* in main text).

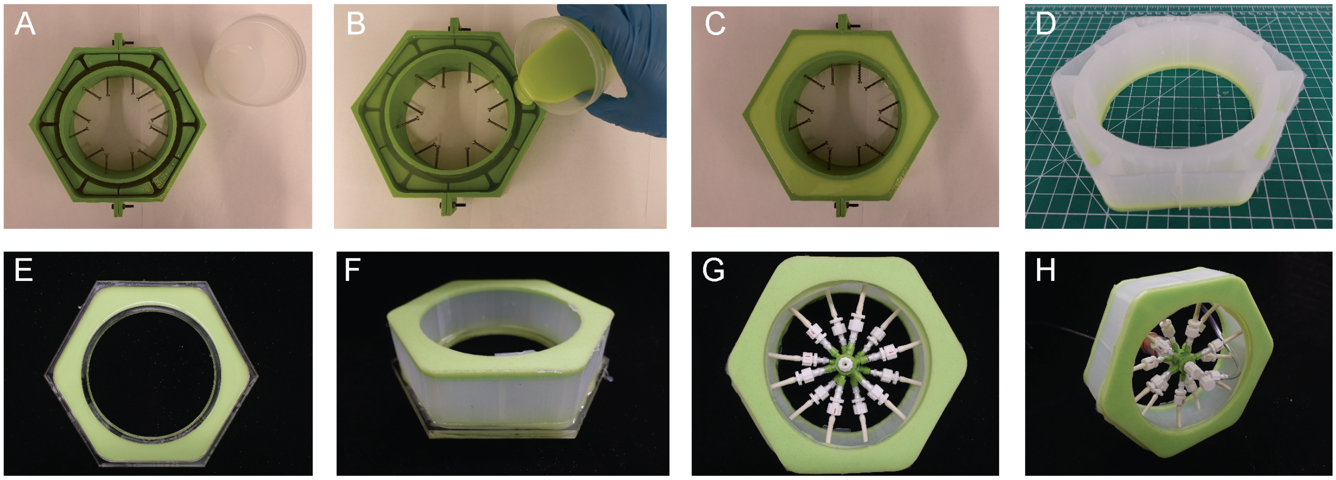

Fig. S6. Fabrication of the rolling robot. Snapshots of the 8 steps required to fabricate the robot.

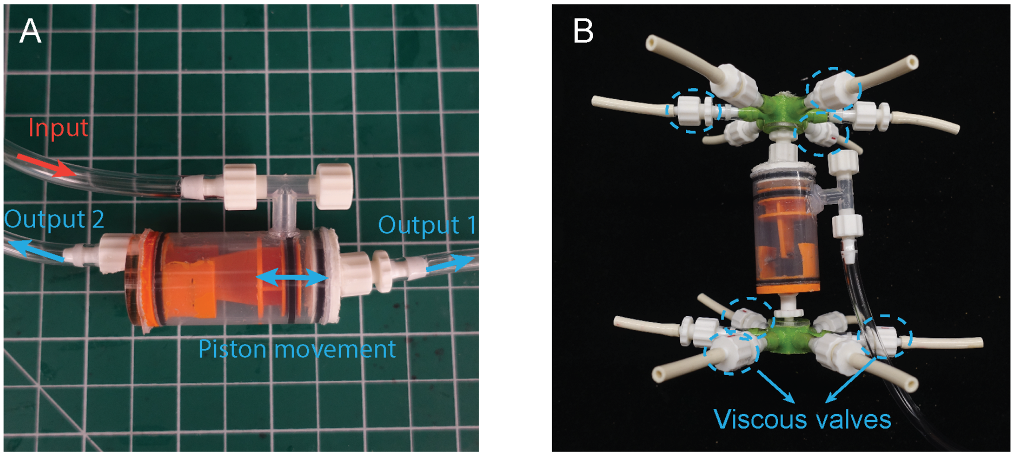

Fig. S7. Connection of the valves for rolling robot. (*A*) Working principle of the modified bistable valve. By changing the position of the piston, we can switch the flow from one circuit to the other. (*B*) The connection between the bistable valve and the two circuits.

2. Experiments

In order to characterize the effect of the valves on the response of the robotic systems, we use pressurized air of the order of a few kPa. To achieve such levels of pressure, we decrease the pressure from the wall air-outlet (∼200 psi) using two pressure regulators (B74G-4AK-AD3-RMN by IMI Norgren Inc and ITV1030 by SMC Cooperation). The first pressure regulator reduces the inlet pressure from 200 psi to about ∼200 kPa, while the second one accurately controls the pressure in the range [0, 200] kPa. Further, to turn on and off the input pressure we used a standard two-way solenoid valves (KVE32PL24FF by Kamoer) to switch on/off the inlet of the system to the atmosphere, so that the actuators could deflate and return to their initial state.

2.1. Characterization of the mechanical valves. To characterize all our mechanical valves, we connect each of them to an extension actuator (Actuator II in Table S1) and monitor the pressure evolution both at the inlet and inside the actuator using two pressure sensors (MPXV7025DP, Freescale Semiconductor Inc - Fig. S8). During the tests the deformation of the actuator is also recorded by a high-resolution camera (SONY EX100V) at a frame rate of 30 fps. The recorded input/output pressure evolution for each valve is reported in Fig. 1 of the main text.

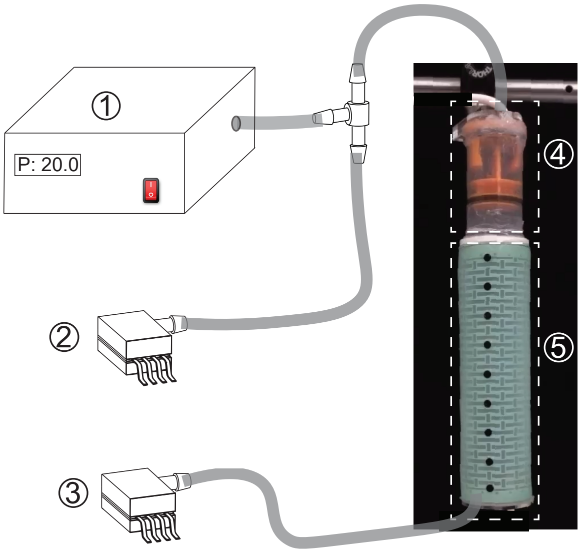

Fig. S8. Characterization of the mechanical valves. Schematic of the test setup used to characterize the valves. (1) Pressure control system. (2) Pressure sensor for the inlet. (3) Pressure sensor for the actuator. (4) Mechanical valve. (5) Actuator.

2.2. Characterization of the kirigami actuators. As part of this study we also experimentally characterize the pressure-volume evolution of each kirigami actuator using the setup shown in Fig. S9A. To decouple the effect of the geometry of the actuators from that of the compressibility of the fluid, we determine the pressure-volume curve by inflating the actuators with water. Further, to eliminate the influence of gravity, we submerge the entire actuator in a water tank. In all our tests, we first fill the actuator with the amount of water corresponding to the initial volume of the cavity. Then, we use a syringe pump (Pump 33DS, Harvard Apparatus) to displace an additional volume of water ΔV into the balloons at 20 mL/min (ensuring quasi-static conditions) and record the pressure using a pressure sensor (MPXV7025DP, Freescale Semiconductor Inc). Since as part of this study we use the kirigami actuators in an unconstrained environment for the robotic arm

and constrained within a tube for the climbing robot, we test them both in unconstrained and constrained conditions. More specifically, we test Actuator I and II under unconstrained conditions and find a liner dependence of the pressure from the supplied volume (Fig. S9*B*). Further, we test Actuator II and III when placed in a tube with diameter of 35 mm (identical to the tube used to test the climbing robot). As shown in Fig. S9*C*, in this case we find a highly non-linear pressure-volume curve, as the pressure abruptly increases when the actuators get in contact with the tube.

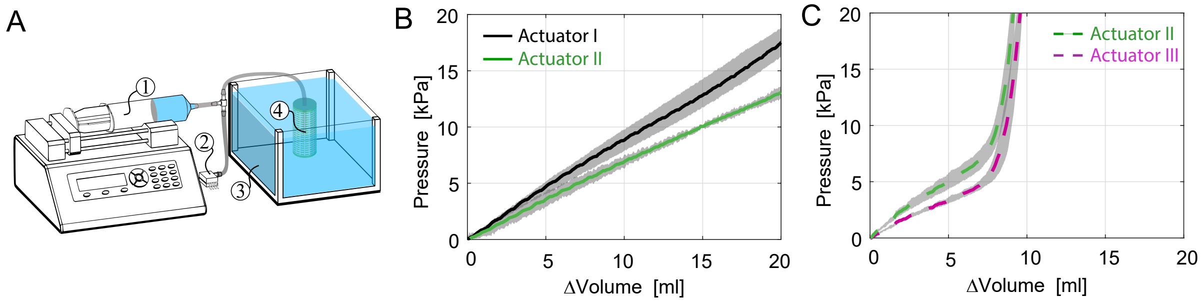

Fig. S9. Pressure-volume characterization of the kirigami actuators. (*A*) Schematic of the test setup used to characterize the pressure-volume relations of actuators. (1) Syringe pump. (2) Pressure sensor. (3) Water tank. (4) Actuator. (*B*) Pressure-volume relations recorded for Actuators I and II when tested in unconstrained conditions. (*C*) Pressure-volume relations recorded for Actuators II and III when tested in constrained conditions.

2.3. Robotic Arm. To characterize the deformation of our robotic arm, we monitor with a high-resolution camera (SONY RX100V) recording at a frame rate of 30 fps (*i*) 17 black circular markers uniformly placed along the length of the kirigami actuators and (*ii*) a white circular marker placed at the end of an acrylic rob connected to the bottom cap. We extract the coordinates of all markers using an open-source digital image correlation and tracking package (3). We then use the coordinates of the white marker to determine the trajectory followed by the robotic arm. Further, we determine the radius R of the circle that best fits the black markers via a direct least-square algorithm (4, 5) and calculate the average curvature of the two actuators as

$$\kappa = 1/R. \quad \text{[S1]}$$

Finally, we note that during the tests the evoultion of the pressure inside the actuators is monitored by pressure sensors (MPXV7025DP, Freescale Semiconductor Inc).

In Figs. S16 and S17 we consider the robotic arm with a viscous valve and one-way viscous valve, respectively, and report the recorded tip trajectory (left), pressure evolution in both actuators (middle) and curvature evolution of both actuators (right) for different pressure inputs.

2.4. Tube climbing robot. To test the climbing robot, we place the system inside a vertically oriented acrylic tube with inner diameter of 35 mm and supply many pressure pulses, while recording its motion using a high-resolution camera (SONY RX100V).

Further, we conduct additional tests to characterize both the frictional force between the expanding actuator and the tube and the axial force exerted by the extending actuator interaction between the robot and the tube, as well as the extension force generate by the extending actuator.

As shown in Fig. S10*A*, to characterize the frictional force, we place an expanding actuator inside a vertically oriented acrylic tube (with inner diameter of 35 mm) and connect it to a motorized translation stage (LTS300/M , ThorLabs) via a nylon thread. During our tests, we inflate the actuator by supplying different amount of pressure ($0 - 6$ kPa) and pull the actuator upward using the linear stage with speed of 0.2 mm/s. The friction force between the tube and actuator is measured using a 10 lb load cell (LSB200 Miniature S-Beam Jr. Load cell, FUTEK Advanced Sensor Technology, Inc.). The recorded evolution of the friction as a function of applied pressure is reported in Fig. 3*C* of the main text.

To measure the axial force generated by the extending actuator, we use the same setup as that use to measure the frictional force except that we use screws to connect the load cell to the linear stage and the actuator(Fig. S10*B*). During our tests, we fix the movement of the linear stage and increase the pressure inside the actuator gradually and record the axial force as a function of the pressure using the load cell. The recorded evolution of the force as a function of applied pressure is reported in Fig. 3*C* of the main text.

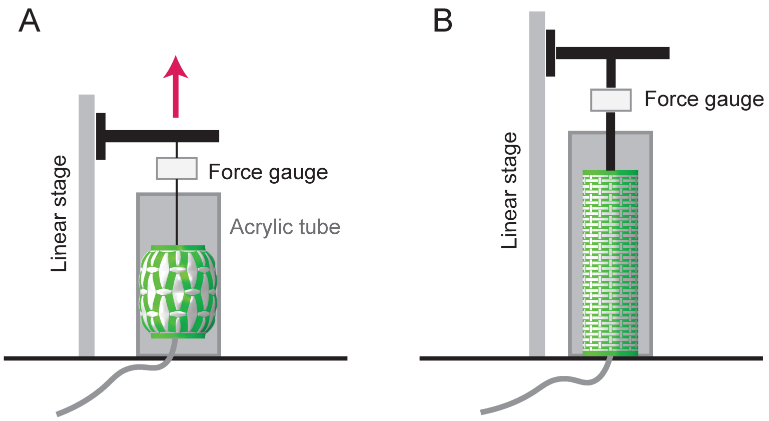

Fig. S10. Schematics of the experimental setups used to measure frictional and extension forces. (*A*) Setup for measuring friction. (*B*) Setup for measuring extension force.

2.5. Rolling robot. To actuate our rolling robot we connect it to our pressure control system (Fig. S8) and supply the pressure profile shown in Fig. S11. Note that the input pressure for each cycle consists of 2 pressure pulses:

- *Pulse 1:* the first pressure pulse ($p_{input} = 15$ kPa for $t_{input} = 3$ s) is provided to rapidly inflate the chambers connected directly to the pressure supplier (snapshot 2 in Fig. 4C of main text). This high pressure pulse accelerate the inflation of the chambers connected directly to the pressure source but has negligible effect on the chambers attached to the viscous valves.
- *Pulse 2:* a moderate pressure pulse ($p_{input} = 10$ kPa for $t_{input} = 67$ s) then follows the high pressure pulse to gradually inflate the chambers attached to the viscous valves. Note that after supplying this second pulse all chambers are inflated (snapshot 4 in Fig. 4C of main text).

When the input pressure is removed, the chambers with no viscous valve deflate instantaneously, while the ones with viscous valves keep the inflated state temporarily and push the robot to rotate further (snapshot 5 in Fig. 4C of main text).

To change the rolling direction of the robot we apply a negative pressure burst with magnitude large enough (∼25 kPa) to change the position of the piston and snap the arch at the same time.

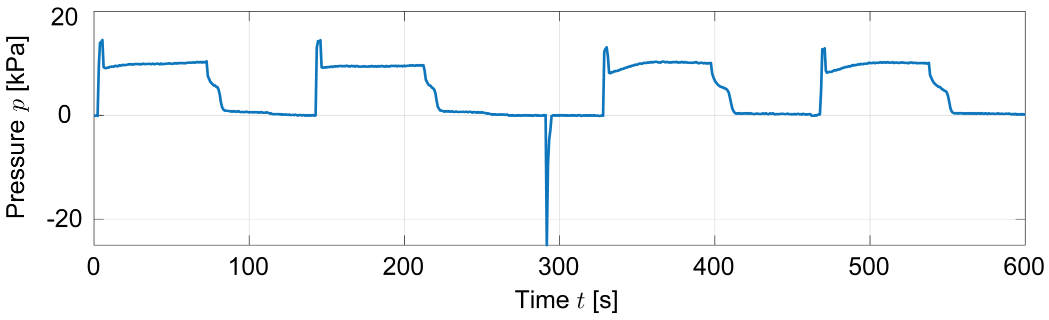

Fig. S11. Pressure supplied to the rolling robot for bidirectional locomotion.

3. Modeling

To get a better understanding of the behavior of the proposed valves, we use numerical analyses. For the viscous valves, we simplify the Navier–Stokes equations to calculate the pressure drop. For the hysteretic and bistable valves, we conduct Finite Element (FE) simulations to characterize the mechanical response of the arches.

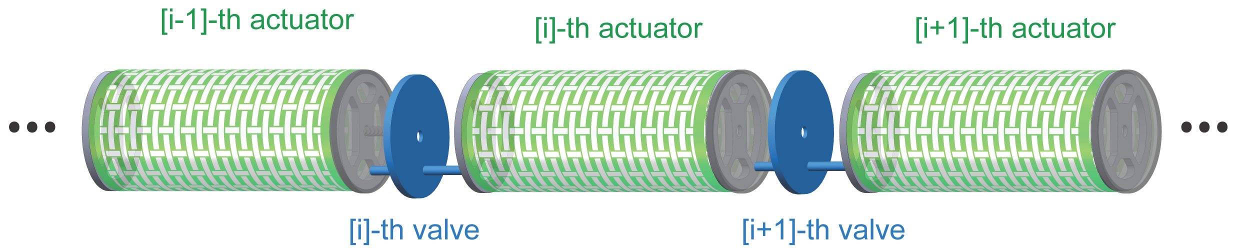

Fig. S12. Schematic of the model for the viscous valves.

3.1. Viscous valves. Our viscous valves comprise narrow tubes to provide a transient pressure difference between adjacent actuators. To predict their response, we use a numerical model recently proposed by Vasios et al (6). More specifically, we focus on the $[i]$-th valve in the actuator that comprises a narrow tube of length $l_{tube,i}$ and diameter $d_{tube,i}$ and assume that

- the tube is rigid and not deformed by the flow;
- the head losses due to friction at the connections between the tube and the actuators can be captured by adjusting its length to $l^{eq}_{tube,i}$;
- the flow is incompressible and laminar in the narrow tube;
- the fluid velocity has the form

$$\mathbf{u} = -\frac{8}{\pi d^2_{tube,i}} \frac{d\tilde{v}_i}{dt} \left[\left(\frac{r}{R_i} \right)^2 - 1 \right] \mathbf{e}_z, \qquad \text{[S2]}$$

  where $\tilde{v}_i = \int_0^t \int_0^{d_{tube,i}/2} \mathbf{u} \cdot \mathbf{e}_z 2\pi r dr dt$ denotes the amount of fluid exchanged through the $[i]$–th tube up to time t and $\mathbf{e}_z$ identifies the tangent vector to the tube;
- the inertia term is negligible, since the tubes are narrow ($l_{tube,i} >> d_{tube,i}$).

Under these assumptions, integration of the Navier–Stokes equations over the volume of the tube yields

$$\frac{d\tilde{V}_i}{dT} + \xi_i (P_i - P_{i-1}) = 0, \quad \text{for} \;\; i = 1, ..., N \qquad \text{[S3]}$$

with

$$\xi_i = \frac{\pi G d^4_{tube,i} t_{max}}{128 \mu v_0 l^{eq}_{tube,i}}, \qquad \text{[S4]}$$

where $\tilde{V}_i = \tilde{v}_i / v_0$ is the normalized volumetric flow through the $[i]$-th tube (v_0 denoting the volume of the largest actuator in the system), $T = t/t_{max}$ is the normalzied time (t_{max} denoting the response

time of the system) and $P_i = p_i/G$ is the normalized pressure in the $[i]$-th actuator (p_i being the pressure inside the $[i]$-th actuator and G being the shear modulus of the material used to fabricate the actuator). Note that $1/\xi_i$ expresses an equivalent resistance that the $[i]$-th valve imposes to fluid flow; when ξ_i is large, high flow rates dV_i/dT are achieved for relatively low pressure differences, whereas when ξ_i is small, the opposite is true.

Next, we connect the amount of fluid exchanged through the valves to the amount of fluid present in the chambers of the actuators. When an incompressible fluid such a water is used to inflate the system, the normalized change in volume for the $[i]$-th actuator, $\Delta V_i = \Delta v_i/v_0$, can be expressed in terms of the volumetric flows exchanged through the two tubes connected to it as

$$\Delta V_i = \tilde{V}_i - \tilde{V}_{i+1}. \qquad \text{[S5]}$$

Differently, when, as in this study, we use a compressible fluid (air) to inflate the robotic system, we focus on the number of moles of fluid exchanged though the valves. Specifically, the change in number of moles of air for the $[i]$-th actuator, Δn_i, can be determined as

$$\Delta n_i = \tilde{n}_i - \tilde{n}_{i+1}, \qquad \text{[S6]}$$

where $\tilde{n}_i$ denotes the number of moles of air exchanged through the $[i]$-th valve. Further, the total number of moles of air in the $[i]$-th actuator at time T can be expressed as

$$n_i(T) = n_i(T=0) + \int_0^T \frac{d\Delta n_i}{dT} dT = n_i(T=0) + \int_0^T \frac{d\left(\tilde{n}_i - \tilde{n}_{i+1}\right)}{dT} dT. \qquad \text{[S7]}$$

Note that, by introducing the ideal gas law n_i and $\tilde{n}_i$ can be written as

$$n_i = \frac{P_i V_i}{R_g \Theta_i}, \quad \tilde{n}_i = \frac{\bar{P}_i \tilde{V}_i}{R_g \bar{\Theta}_i}, \qquad \text{[S8]}$$

where V_i denotes the volume for the $[i]$-th actuator, R_g is the ideal gas constant and Θ is the temperature. Moreover,

$$\bar{P}_i(T) = \begin{cases} P_{i-1} & \text{for } \frac{d\tilde{V}_i}{dT} \geq 0, \\ P_i & \text{for } \frac{d\tilde{V}_i}{dT} < 0, \end{cases}$$

and

$$\bar{\Theta}_i(T) = \begin{cases} \Theta_{i-1} & \text{for } \frac{d\tilde{V}_i}{dT} \geq 0, \\ \Theta_i & \text{for } \frac{d\tilde{V}_i}{dT} < 0. \end{cases}$$

Note that if the air flows from the $[i-1]$-th actuator to $[i]$-th acuator, the pressure and temperature inside the $[i-1]$-th actuator should be used to calculate $\tilde{n}_i$, whereas when the air flows from the $[i]$-th actuator to $[i-1]$-th acuator, $\tilde{n}_i$ is determined based on the pressure and temperature inside the $[i]$-th actuator.

If we assume the actuation to be an isothermal process (i.e. we assume that the temperature of the air inside the actuators is constant), substitution of Eqs. (S8) into Eq. (S7) yields

$$P_i(T)V_i(T) = P_i(0)V_i(0) + \int_0^T \bar{P}_i(T)\frac{d\tilde{V}_i(T)}{dT}\mathrm{d}T - \int_0^T \bar{P}_{i+1}(T)\frac{d\tilde{V}_{i+1}(T)}{dT}\mathrm{d}T, \qquad \text{[S9]}$$

where $V_i(0)$ is the initial volume of the $[i]$-th actuator and $P_i(0)$ is the initial pressure inside the $[i]$-th actuator (i.e. atmospheric pressure $P_i(0) = 101.3$ kPa). Finally, by substituting Eq. (S3) into

Eq. (S9), we obtain

$$P_i(T)V_i(T) = P_i(0)V_i(0) - \int_0^T \xi_i \bar{P}_i(T)\left[P_i(T) - P_{i-1}(T)\right)] \,\mathrm{d}T + \int_0^T \xi_{i+1}\left[\bar{P}_{i+1}(T)(P_{i+1}(T) - P_i(T))\right] \mathrm{d}T, \quad \text{[S10]}$$

For a system comprising N fluidic actuators interconnected via viscous valves Eq. (S10) results in a system of N coupled differential equations, which given a pressure-volume relationship for the actuators that can be numerically solved to determine the normalized change in volume for the $[i]$-th actuator as a function of time. Finally, we note that for the first and last tube in the array Eq. (S10) needs to be modified as

$$P_1(T)V_1(T) = P_1(0)V_1(0) - \int_0^T \xi_1 \bar{P}_1(T)\left[P_1(T) - P_{input}(T)\right)] \,\mathrm{d}T + \int_0^T \xi_2\left[\bar{P}_2(T)(P_2(T) - P_1(T))\right] \mathrm{d}T, \quad \text{[S11]}$$

and

$$P_N(T)V_N(T) = P_N(0)V_N(0) - \int_0^T \xi_N \bar{P}_N(T)\left[P_N(T) - P_{N-1}(T)\right)] \,\mathrm{d}T, \quad \text{[S12]}$$

to account for the pressure input and the end of the array.

Individual valves. For the simple setup considered in Fig. 1*D* of the main text (comprising a rigid chamber and an extension kirigami actuator separated by a viscous valve), Eq. (S10) specializes to

$$P(T)V(T) = P(0)V(0) - \int_0^T \xi \bar{P}(T)(P(T) - P_{input}(T))\mathrm{d}T, \quad \text{[S13]}$$

where P and $\tilde{V}$ denote the volume and pressure of the actuator and $P_{input}(t)$ is the normalized input pressure supplied to the rigid chamber

$$P_{input}(T) = \begin{cases} p_{input}/G & \text{for } T \leq T_{input}, \\ 0 & \text{for } T > T_{input}. \end{cases} \quad \text{[S14]}$$

Note P and V are not independent, as they follow the experimentally measured pressure-volume relationship shown in Fig. S9. To estimate ξ for a given valve we compare the time evolution of pressure measured in our experiments with the numerical prediction obtained by numerically solving Eq. (S13) with the pressure-volume relationship reported in Fig. S9*B* (Actuator II). We find excellent agreement between experimental results and numerical predictions when choosing (see Fig. S13)

- $\xi = 14.2$ for a viscous valve with $d_{tube} = 0.21$ mm;
- $\xi = 14.2$ for a one-way viscous valve with $d_{tube} = 0.21$ mm and flow in the forward direction
- $\xi = 716.2$ for a one-way viscous valve with $d_{tube} = 0.21$ mm and flow in the backward direction

Note that these values for ξ are also summarized in Table S2.

Robotic arm. For the robotic arm considered in Fig. 2 of the main text (which comprises two bending kirigami actuators connected through a viscous valve with $d_{tube} = 0.21$ mm), Eq. (S10) specializes to

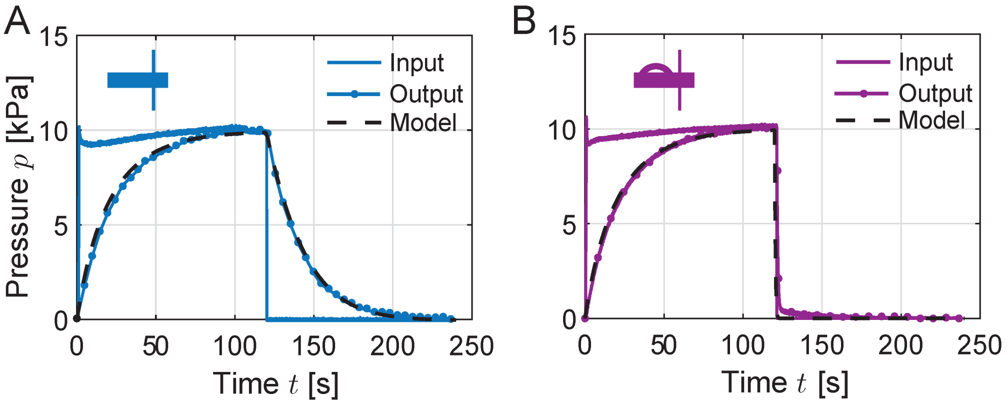

Fig. S13. Model vs. experiments. (*A*) Comparison between experimental results (blue line) and model predictions (black line) for a viscous valve with tube length $l_{tube} = 25$ mm and internal diameter $d_{tube} = 0.21$ mm. (*B*) Comparison between experimental results (purple line) and model predictions (black line) for a one-way viscous valve with tube length $l_{tube} = 25$ mm and internal diameter $d_{tube} = 0.21$ mm. The equivalent conductance use in the mechanical valves are reported in Table S2.

	Viscous valve ($d_{tube} = 0.21$ mm)	One-way viscous valve ($d_{tube} = 0.21$ mm) [forward direction]	One-way viscous valve ($d_{tube} = 0.21$ mm) [backward direction]
ξ_i	14.2	14.2	716.2

Table S2. Equivalent conductance of the viscous valves.

$$\begin{cases} P_1(T)V_1(T) = P_1(0)V_1(0) - \int_0^T \xi_1 \bar{P}_1(T)(P_1(T) - P_{input}(T))\mathrm{d}T + \int_0^T \xi_2 \bar{P}_2(T)(P_2(T) - P_1(T))\mathrm{d}T, \\ P_2(T)V_2(T) = P_2(0)V_2(0) - \int_0^T \xi_2 \bar{P}_2(T)(P_2(T) - P_1(T))\mathrm{d}T, \end{cases} \quad [S15]$$

where the subscripts 1 and 2 refer to the top and bottom actuator, respectively. Further, ξ_1 and ξ_2 denote the equivalent resistance encountered by the fluid between the input source and the top actuator and the equivalent resistance imposed by the viscous valve, respectively. Note that the values for ξ_2 are reported in Table S2, whereas for the connection between the input source and the first actuator we use $\xi_1 = 472.7$. Finally, to connect P and V, we use the experimentally measured pressure-volume relationship shown in Fig. S9*B* (Actuator I). As shown, in Figs. S16 and S17 the model nicely capture the response of the arm with both a viscous and one-way viscous valve for a wide range of pressure inputs.

Climbing robot. For the climbing robot considered in Fig. 3 of the main text (which comprises three bending kirigami actuators connected through a viscous valve with $d_{tube} = 0.21$ mm and a one-way viscous valve with $d_{tube} = 0.21$ mm), Eq. (S10) specializes to

$$\begin{cases} P_1(T)V_1(T) = P_1(0)V_1(0) - \int_0^T \xi_1 \bar{P}_1(T)(P_1(T) - P_{input}(T))\mathrm{d}T + \int_0^T \xi_2 \bar{P}_2(T)(P_2(T) - P_1(T))\mathrm{d}T, \\ P_2(T)V_2(T) = P_2(0)V_2(0) - \int_0^T \xi_2 \bar{P}_2(T)(P_2(T) - P_1(T))\mathrm{d}T + \int_0^T \xi_3 \bar{P}_3(T)(P_3(T) - P_2(T))\mathrm{d}T, \\ P_3(T)V_3(T) = P_3(0)V_3(0) - \int_0^T \xi_3 \bar{P}_3(T)(P_3(T) - P_2(T))\mathrm{d}T, \end{cases} \quad [S16]$$

where the subscripts 1, 2 and 3 refer to the bottom, middle and top actuators. Further, ξ_1 is the equivalent resistance between the input source and the bottom actuator, ξ_2 denotes the equivalent resistances of the one-way viscous valve and ξ_3 represent the equivalent resistances of the viscous valve. Note that the values for ξ_2 and ξ_3 are reported in Table S2, whereas for the connection

between the input source and the first actuator we use $\xi_1 = 472.7$. Finally, to connect P and V, we use the experimentally measured pressure-volume relationship shown in Fig. S9C. As shown in Fig. S20. we find that the model (dashed lines) agree well with the experimental measurements (solid lines) for all considered pressure inputs.

3.2. Snapping arch. To identify the geometric features resulting either in a snapping or in a bistable arch, we perform Finite Element (FE) analyses using the commercial package ABAQUS 2019/Standard. In our analyses we consider plates with width $w_{plate} = 5$ mm, length $l_{plate} \in [17, 19.5]$ mm, thickness t_{plate} equal to either 0.05 mm or 0.075 mm and mounting angle $\theta_{plate} \in [0°, 60°]$. We discretize the plate using four-node general-purpose shell elements with reduced integration and hourglass control (S4R element type). Further, since plasticity has little effect on the observed phenomena, we capture the material behavior using a linear elastic material model (with Young's modulus $E = 170$ GPa and Poisson's ratio $\nu = 0.3$). We then simulate the response of the arch conducting non-linear static simulations under displacement control and dynamic implicit simulations under load control. The simulations consist of two steps

- **Step 1:** We buckle a flat plate to an arch by applying an in-plane compression displacement $d_{in} = (l_{plate} - w)/2$ ($w = 16$ mm being the distance between the two slits of the holder - Fig. S14B) and a rotation angle θ_{plate} to both end of the arch.
- **Step 2:** We fix the displacement and rotation at both ends of the arch. For displacement control simulations we apply an out-of-plane displacement d_{out} at the center of the arch (see Fig. S14A)) and record the reaction force F. For the load control simulations, we apply a force F at the center of the arch (see Fig. S14B) and record the displacement d_{out}. Note that the force F can be expressed as a function of the applied pressure as

$$F = p\pi r^2, \qquad [S17]$$

where r is the radius of the chamber.

In Fig. S14C, we report the numerically predicted evolution of the equivalent pressure p as a function of the out-of-plane displacement d_{out} for an arch with $\theta_{plate} = 45°$, $l_{plate} = 17.5$ mm and $t_{plate} = 0.075$ mm. The continuous line represents the FE results of the arch under displacement control (by applying a displacement d_{out} at the center of the arch). The non-monotonic behavior of the arch is clearly visible. However, in our mechanical valves the flow pressurizes the piston, which transfers the force to the central point of the arch. Hence, the boundary conditions of the experiments are different from those of the simulations under displacement control. To quantify the effect of such boundary conditions, we also report the response obtained from our load control simulations (black dashed curves in Fig. S14C). In this case we can see that the pressure-displacement relation of the arch exhibits a linear regime at the beginning of the loading step. Then, as the pressure increases to a certain value p^*_{on}, the central point of the arch jumps from positions I to II (characterized by a plateau on the curve) and, in turn, changes the position of the piston and switches on the valve. Furthermore, when we reduce the pressure, the arch can snap back at pressure p^*_{off} from positions III to IV and switch off the valve.

It is worth noticing that the model results have to be corrected by taking into account the contribution of the friction force between the piston and the chamber. We measure this force to be $F_{friction} = 2.2$ N and always opposed to the moving direction of the piston. Therefore the real

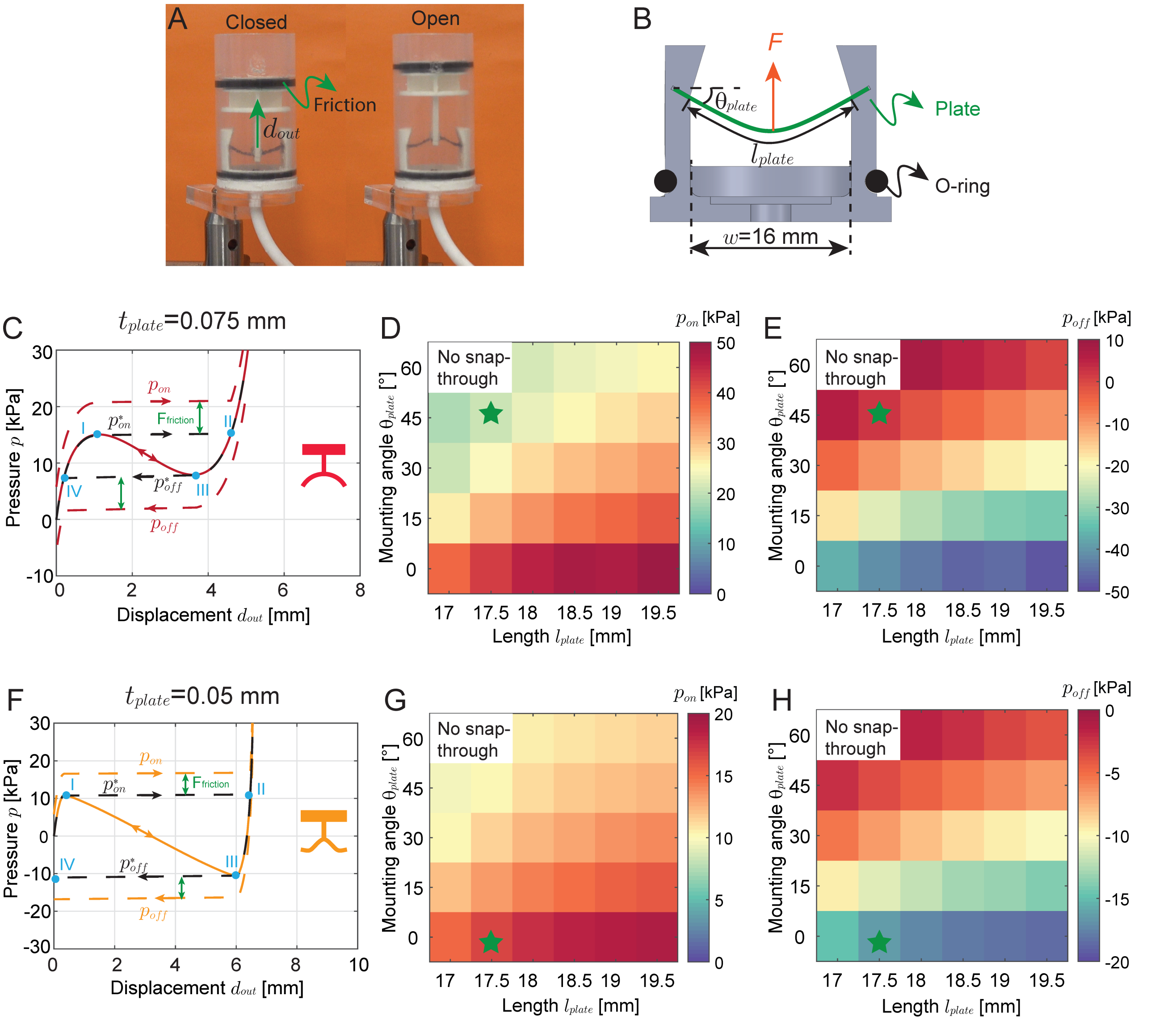

Fig. S14. FE simulations for the hysteretic and bistable valves. (*A*) Experimental snapshots of a bistable valve in the closed and open states. (*B*) Schematic of a 3D printed holder for the plate. The mechanical behavior of the valve can be tuned by changing the mounting angle (θ_{plate}), length (l_{plate}) and thickness (t_{plate}) of the plate (we fix the width of the plate $w_{plate} = 5$ mm throughout this study). (*C*) Mechanical response of an hysteretic valve with $\theta_{plate} = 45$ °, $l_{plate} = 17.5$ mm and $t_{plate} = 0.075$ mm. Continuous red line and black dashed lines represent the FE results of the plate under displacement control and force control, respectively. Red dashed lines represent the behavior of the valve with consideration of the friction between the piston and the chamber. The on/off pressures of the valve are $p_{on} = 20.8$ kPa and $p_{off} = 2.1$ kPa, respectively. (*D*,*E*) Phase diagrams of (*D*) p_{on} and (*E*) p_{off} for a valve with $t_{plate} = 0.075$ mm as a function of θ_{plate} and l_{plate}. (*F*) Mechanical response of a bistable valve with $\theta_{plate} = 0°$, $l_{plate} = 17.5$ mm and $t_{plate} = 0.05$ mm. Continuous orange line and black dashed lines represent the FE results of the plate under displacement control and force control, respectively. Orange dashed lines represent the behavior of the valve with consideration of the friction between the piston and the chamber. The on/off pressures of the valve are $p_{on} = 16.3$ kPa and $p_{off} = -16.3$ kPa, respectively. (*G*,*H*) Phase diagrams of (*G*) p_{on} and (*H*) p_{off} for a valve with $t_{plate} = 0.05$ mm as a function of θ_{plate} and l_{plate}.

response of the valve can be obtained by shifting the black dashed line up (for the loading curve) or down (for the unloading curve) by the amount of the equivalent pressure generated by the friction force (see red dashed lines in Fig. S14*C*). As a result, the corrected on/off pressure of the valve reads

$$p_{on} = p^*_{on} + \frac{F_{friction}}{\pi r^2}, \qquad p_{off} = p^*_{off} - \frac{F_{friction}}{\pi r^2}. \qquad \text{[S18]}$$

In order to design a valve with a prescribed on/off pressure, in Figs. S14*D* and *E* we report the phase diagrams of p_{on} and p_{off} for a valve with $t_{plate} = 0.075$ mm as a function of the mounting

angle θ_{plate} and length l_{plate}. Instructed by the model, we choose $\theta_{plate} = 45°$ and $l_{plate} = 17.5$ mm as design parameters, which enables the on/off switch of the valve at 20.8 kPa and 2.1 kPa, respectively.

For the bistable valve, we reduce the thickness of the plate to $t_{plate} = 0.05$ mm in order to operate the valve at around 20 kPa. Similarly, we report the response of the bistable valve (with $\theta_{plate} = 0°$ and $l_{plate} = 17.5$ mm) in Fig. S14*F* and the phase diagrams for p_{on} and p_{off} for a valve with thickness $t_{plate} = 0.05$ mm in Figs. S14*G* and *H*, respectively. For the rolling robot, we choose $\theta_{plate} = 0°$ and $l_{plate} = 17.5$ mm as design parameters of the bistable valve, which enable the on/off switch of the valve at 16.3 kPa and −16.3 kPa, respectively.

Please note that under load control the simulation of the snapping arch beyond point I or III often incurs in convergence problems (Fig. S14*C*), providing incomplete information about the response of the arch. However, the local maximum and minimum pressures for the displacement control curves are identical to those for the load control curves. Therefore the p^*_{on} and p^*_{off} values can be extracted from the displacement control simulations. In turn, we can calculate p_{on} and p_{off} for the phase diagrams by correcting these results to take into account friction.

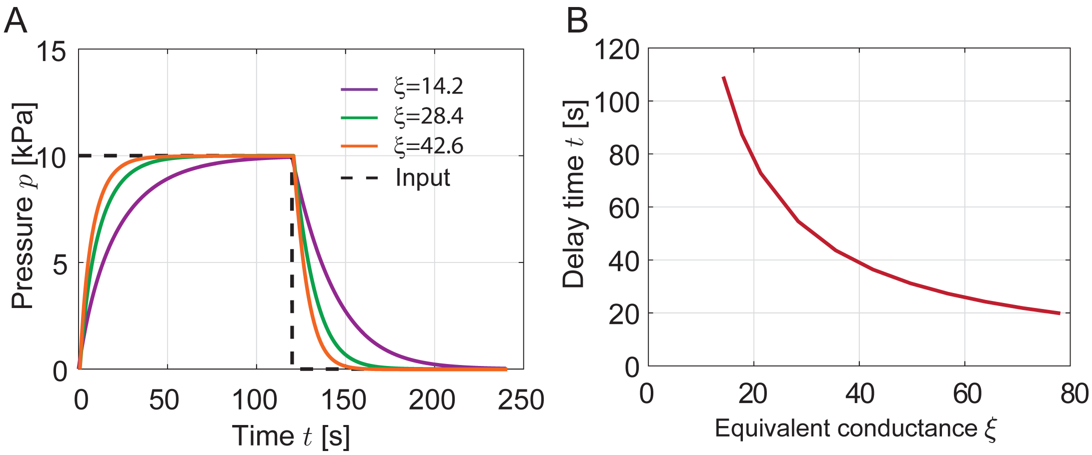

Fig. S15. Effect of the equivalent conductance ξ on the response of our viscous valves. (*A*) Numerically predicted pressure evolution at the inlet and outlet of a viscous valve characterized by ξ =14.2, 28.4 and 42.6 when connected to the extension actuator considered in Fig. 1c. (*B*) Numerically predicted delay time between the input and output (i.e. the time that it takes p_{output} to reach p_{input}) as a function of ξ.

4. Additional Results

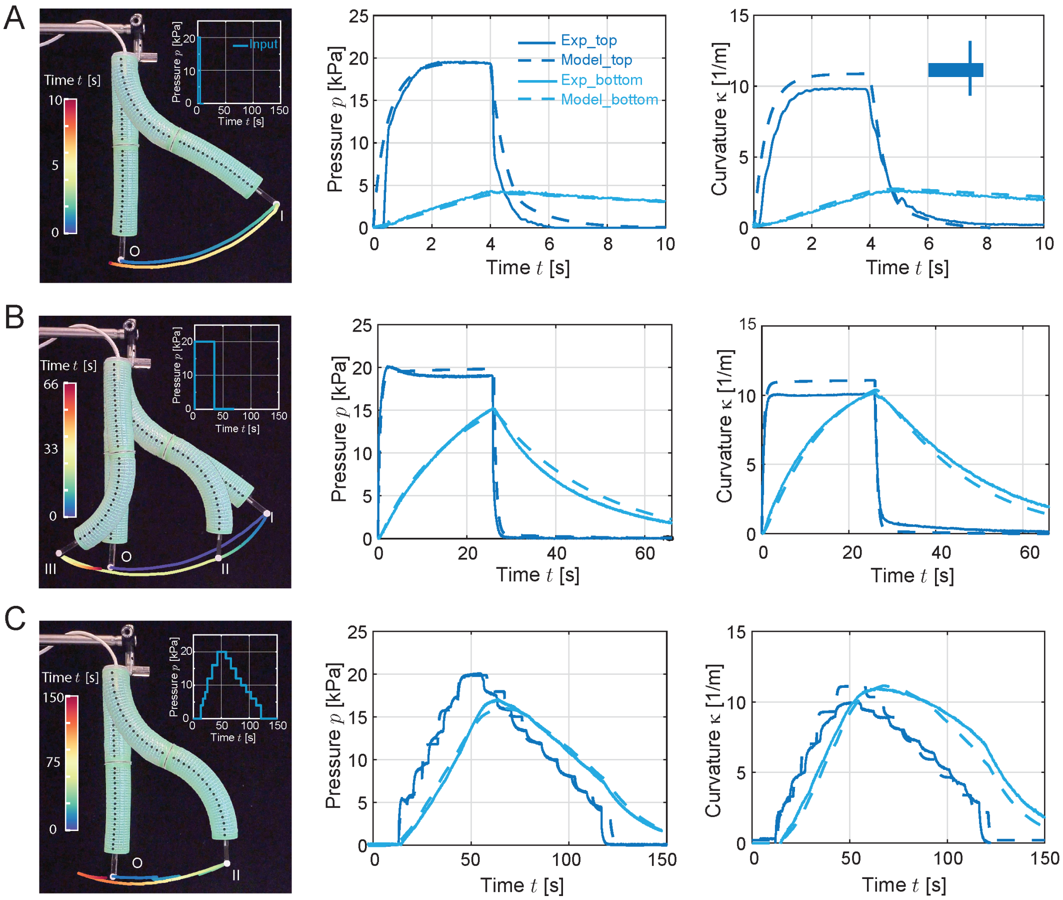

Fig. S16. Robotic arm comprising two bending actuators and a viscous valve. Multiple trajectories of the robotic arm are realized upon application of (*A*) a short rectangular pressure pulse ($p_{input} = 20$ kPa for $t_{input} = 4$ s), (*B*) a long rectangular pressure pulse ($p_{input} = 20$ kPa for $t_{input} = 26$ s) and (*C*) a gradually varied input pressure profile. Our model can accurately predict the pressure and curvature evolution of each actuator.

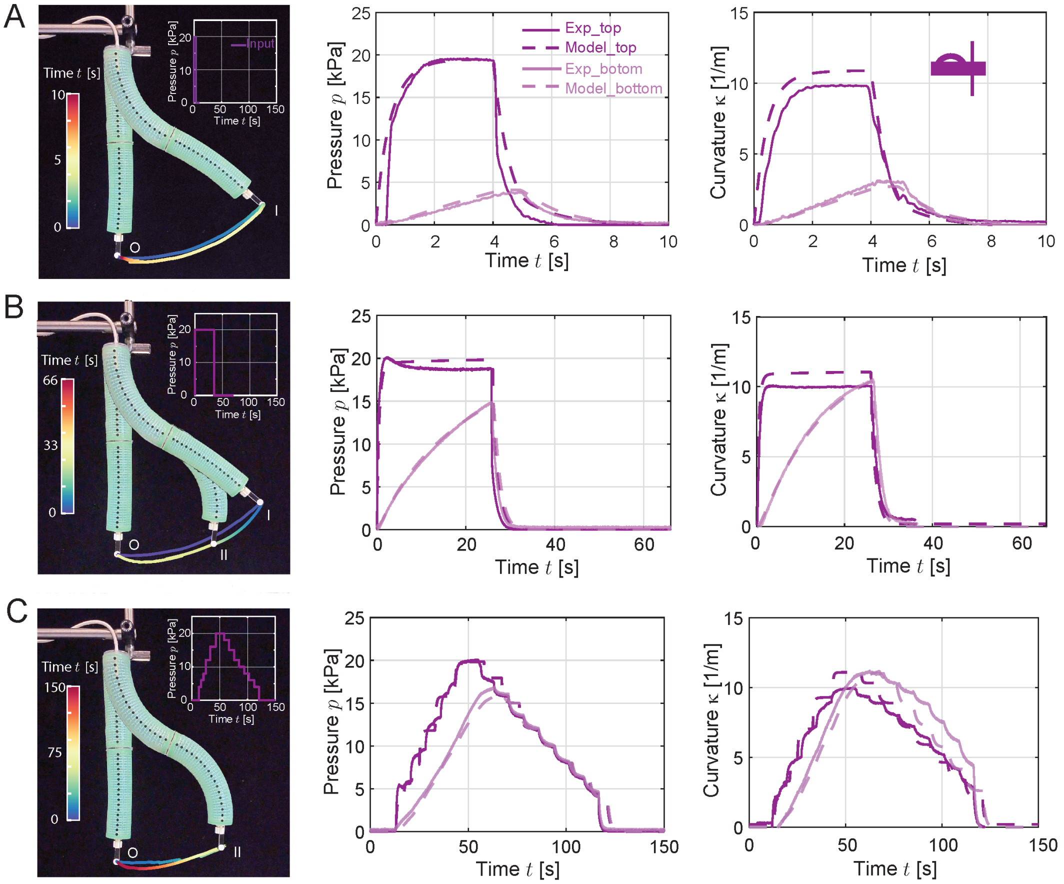

Fig. S17. Robotic arm comprising two bending actuators and a one-way viscous valve. Multiple trajectories of the robotic arm are realized upon application of (*A*) a short rectangular pressure pulse ($p_{input} = 20$ kPa for $t_{input} = 4$ s), (*B*) a long rectangular pressure pulse ($p_{input} = 20$ kPa for $t_{input} = 26$ s) and (*C*) a gradually varied input pressure profile. Our model can accurately predict the pressure and curvature evolution of each actuator.

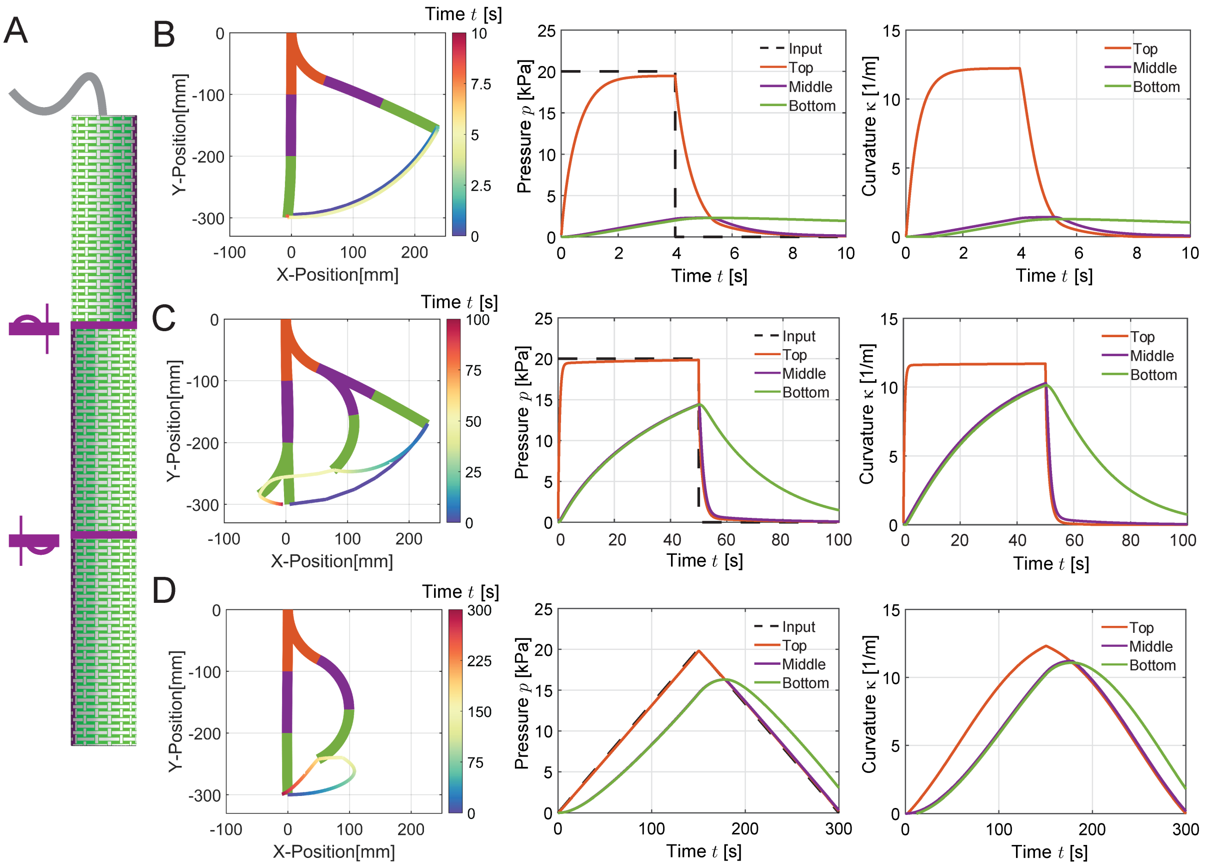

Fig. S18. Numerical results of a robotic arm comprising three bending actuators and two one-way viscous valves. (*A*) Schematic of the robotic arm. (*B*-*D*) Numerical results for the robotic arm upon application of (*B*) a short rectangular pressure pulse ($p_{input} = 20$ kPa for $t_{input} = 4$ s), (*C*) a long rectangular pressure pulse ($p_{input} = 20$ kPa for $t_{input} = 50$ s) and (*D*) a gradually varied input pressure profile.

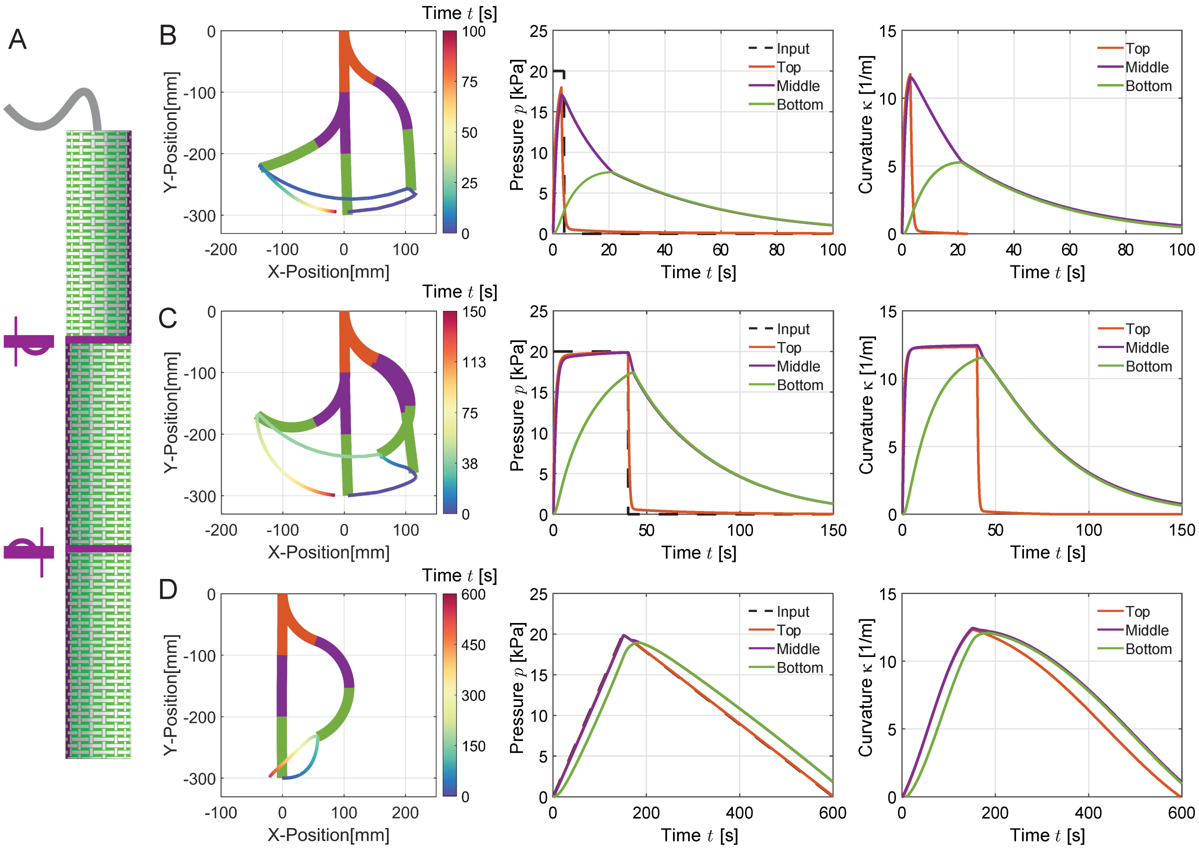

Fig. S19. Numerical results of a robotic arm comprising three bending actuators and two one-way viscous valves. (*A*) Schematic of the robotic arm. (*B-D*) Numerical results for the robotic arm upon application of (*B*) a short rectangular pressure pulse ($p_{input} = 20$ kPa for $t_{input} = 4$ s), (*C*) a long rectangular pressure pulse ($p_{input} = 20$ kPa for $t_{input} = 40$ s) and (*D*) a gradually varied input pressure profile. Note that this robotic arm is different from the one in Fig. S18 because of the flipped configurations of both viscous valves.

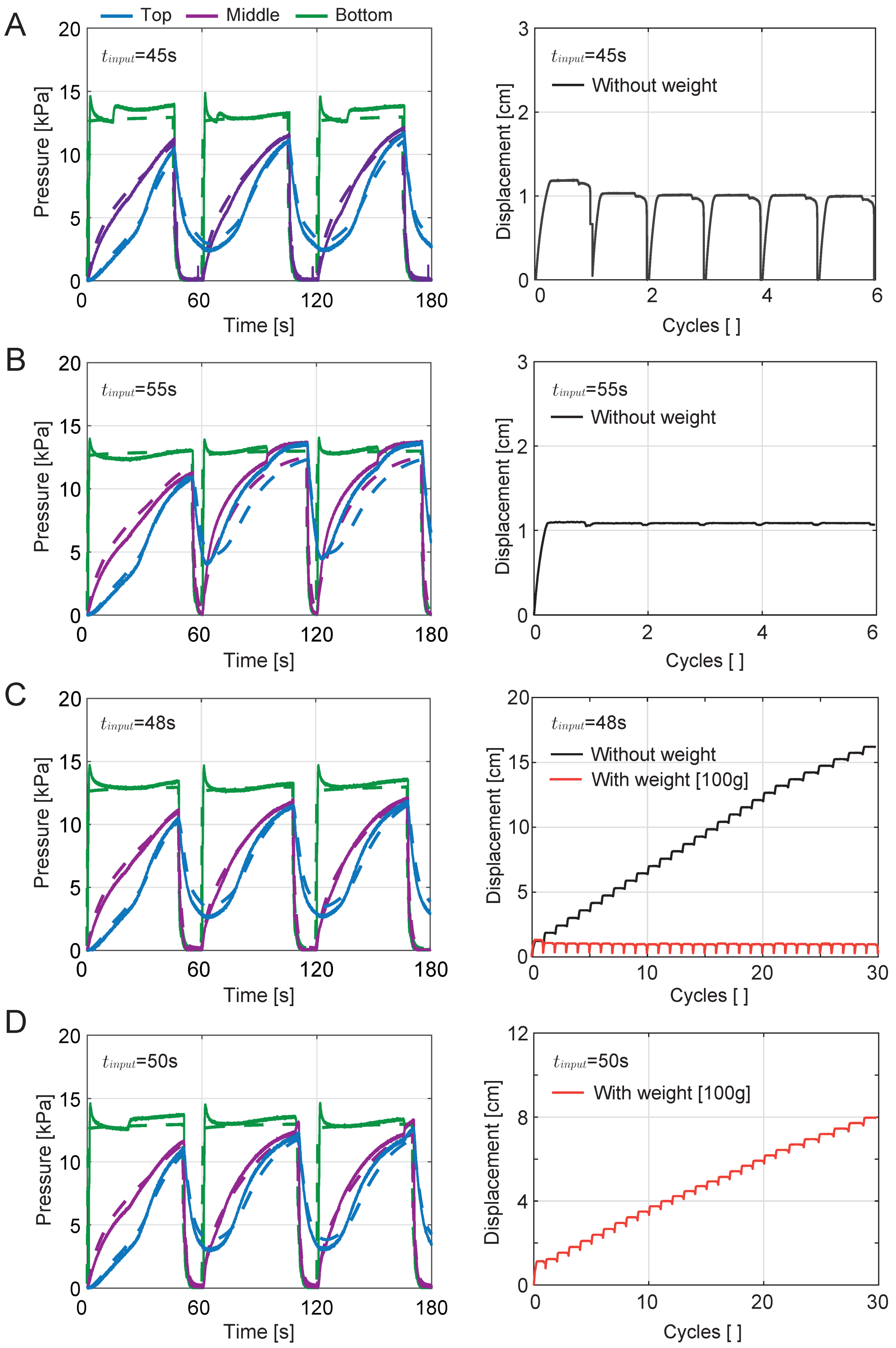

Fig. S20. Pressure evolution and displacements of the climbing robot upon application of a wide range of pressure inputs. (*A*) The robot falls down at the end of each cycle with $t_{input} = 45$ s. (*B*) The robot can not move upwards after the first cycle with $t_{input} = 55$ s. (*C*) The robot climbs up to 16 cm in 30 cycles but can not climb with additional weight (100 g) for $t_{input} = 48$ s. (*D*) The robot climbs up to 8 cm in 30 cycles with additional weight (100 g) with $t_{input} = 50$ s.

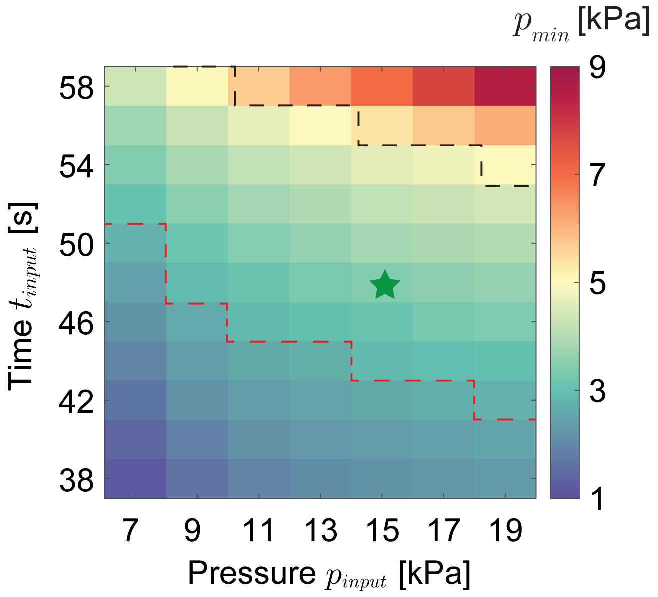

Fig. S21. Phase diagram of the minimum pressure inside the climbing robot's top actuator when a hysteretic valve is embedded into it. The embedded hysteretic valve affects the cavity of the top actuator and, in turn, the pressure-volume evolution. The area of the diagram that is bounded by the two dashed lines identifies the input parameters for which the robot will achieve climbing. Guided by our model, we choose $p_{input} = 48$ s and $p_{input} = 15$ kPa as the input for the grasping tests.

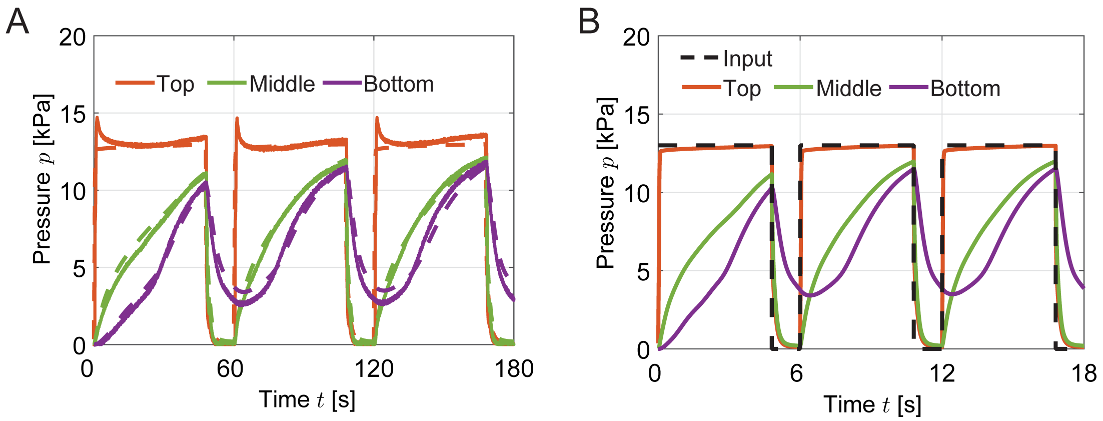

Fig. S22. Effect of the equivalent conductance of both the pump and the valves on actuation speed. (A) Pressure evolution inside the three actuators for the climbing robot considered in Fig. 3 of the main text. (B) Numerically predicted pressure evolution inside the three actuators for a climbing robot for which the equivalent conductance ξ of both the pump and the valves is increased by 10 times.

Movie S1. Characterization of the mechanical valves.

To demonstrate the capabilities of our mechanical valves, we connect each valve to an extending actuator and compare the pressure evolution at the inlet and outlet of the valve.

Movie S2. Robotic arm with different trajectories.

The robotic arm consists of two bending actuators and a viscous valve (or one-way viscous valve). It is capable of achieving multiple trajectories with a single pressure input.

Movie S3. Tube climbing robot.

The climbing robot consists of two expanding actuators, an extending actuator, a viscous valve and a one-way viscous valve. It can climb inside a pipe carrying two times its own weight. When a threshold valve and a gripper are integrated in the robot, the robot can also grasp an object and pull it down. All the movements are achievable with a single pressure input.

Movie S4. Rolling robot.

The hexagonal rolling robot comprises 12 inflatable chambers and six viscous valves arranged into two circuits. A bistable valve activates one circuit at a time, enabling the robot to successfully navigate in both directions with a single input.

References

1. Jin L, Forte AE, Deng B, Rafsanjani A, Bertoldi K (2020) Kirigami-inspired inflatables with programmable shapes. *Advanced Materials* 32(33):2001863.
2. Ilievski F, Mazzeo AD, Shepherd RF, Chen X, Whitesides GM (2011) Soft robotics for chemists. *Angewandte Chemie* 123(8):1930–1935.
3. Senn M (2021) Digital image correlation and tracking: Calculate displacement, strain and stress from image sequences. *MATLAB Central File Exchange.* https://www.mathworks.com/matlabcentral/fileexchange/50994-digital-image-correlation-and-tracking.
4. Pratt V (1987) Direct least-squares fitting of algebraic surfaces. *ACM SIGGRAPH computer graphics* 21(4):145–152.
5. Chernov N (2021) Circle fit (pratt method). *MATLAB Central File Exchange.* https://www.mathworks.com/matlabcentral/fileexchange/22643-circle-fit-pratt-method.
6. Vasios N, Gross AJ, Soifer S, Overvelde JT, Bertoldi K (2020) Harnessing viscous flow to simplify the actuation of fluidic soft robots. *Soft Robotics* 7(1):1–9.
